# Prediction of performance and emission features of diesel engine using alumina nanoparticles with neem oil biodiesel based on advanced ML algorithms

**DOI:** 10.1038/s41598-025-97092-2

**Published:** 2025-04-12

**Authors:** M. S. Aswathanrayan, N. Santhosh, Srikanth Holalu Venkataramana, Kurugundla Sunil Kumar, Sarfaraz Kamangar, Amir Ibrahim Ali Arabi, Sameer Algburi, Osamah J. Al-sareji, A. Bhowmik

**Affiliations:** 1 Department of Mechanical Engineering, ICEAS, Bangalore, Karnataka India; 2 Department of Mechanical Engineering, MVJ College of Engineering, Bangalore, 560 067 India; 3 Department of Aeronautical Engineering, NitteMeenakshi Institute of Technology, Yelahanka, Bangalore, Karnataka 560 064 India; 4 Department of Marine Engineering, Faculty of Engineering, Sri Venkateswara College of Engineering, Pennalur, Sriperumbudur, Tamil Nadu, 602117 India; 5 Mechanical Engineering Department, College of Engineering, King Khalid University, 61421 Abha, Saudi Arabia; 6 Al-Kitab University, Kirkuk, 36015 Iraq; 7 Faculty of Engineering, University of Pannonia, Egyetem Str. 10, Veszprém H, 8200 Hungary; 8 Centre for Research Impact and Outcome, Chitkara University Institute of Engineering and Technology, Chitkara University, Rajpura, Punjab 140401 India; 9 Department of Mechanical Engineering, Graphic Era (Deemed to Be University), Clement Town, Dehradun, Uttarakhand 248002 India

**Keywords:** Linear regression, Decision tree, Random forest, Nanoparticles, Exhaust gas temp, Biofuels, Fossil fuels, Renewable energy, Mechanical engineering

## Abstract

The growing need for sustainable energy sources and stricter environmental regulations necessitate the development of alternative fuels with lower emissions and improved performance. This study addresses these challenges by optimizing the performance and emission characteristics of a single-cylinder diesel engine powered by neem oil biodiesel blends enhanced with alumina nanoparticlesusing the powerful desirability-based optimization. Neem oil, a non-edible feedstock, was selected to avoid competition with food resources, while alumina nanoparticles were utilized for their catalytic properties to enhance combustion efficiency. The process involved experimental evaluation of biodiesel blends (B10, B20, and B30) combined with alumina nanoparticles at concentrations of 100 ppm, 150 ppm, and 200 ppm using a design of experiments approach. With the engine running at maximum load of 100% and an aluminum oxide concentration of 100 parts per million, the optimal fuel mix comprises of 89.85% diesel and 30% biodiesel. The lowest brake-specific fuel consumption of 0.45 kg per kilowatt-hour that the optimization produced points to effective fuel use. With a little variance of 3.33%, the brake thermal efficiency was maximized at 38.18%, quite near to the validation result of 37.89%. The alumina nanoparticles enhanced combustion through improved fuel atomization and oxidation due to their high surface area and catalytic effects. To further validate the effectiveness of RSM, the results are compared with the performance of several advance machine learning algorithms, including linear regression, decision tree, and random forest. The random forest model demonstrated the highest predictive accuracy for performance (test R^2^ = 0.9620, Test MAPE = 3.6795%), making it the most reliable statistical approach for predicting BSFC compared to linear regression and decision Tree models. The random forest model also outperformed other approaches in predicting emissions, achieving the highest accuracy with a test R^2^ of 0.9826 and the lowest test MAPE of 9.3067%.This integrated experimental and predictive approach provided a robust framework for optimizing biodiesel formulations, identifying the ideal combination of biodiesel blend ratio and nanoparticle concentration. The findings highlight the potential of neem oil biodiesel blends enhanced with alumina nanoparticles to achieve a sustainable balance between improved engine performance and reduced emissions in CI engines.

## Introduction:

The growing concern over depleting fossil fuel reserves and escalating environmental pollution has accelerated the search for sustainable and eco-friendly alternatives^1^. Biodiesel, derived from renewable feedstocks, has emerged as a promising substitute for conventional diesel^2,3^. Among various biodiesel sources, neem oil, a non-edible and abundantly available feedstock, offers significant potential due to its high yield and minimal competition with food crops^4,5^. However, challenges such as lower thermal efficiency and higher nitrogen oxide (NOx) emissions limit its widespread application. To overcome these issues, researchers have explored various strategies, including the incorporation of nanoparticles as additives to enhance combustion and emission characteristics^6,7^. The nano particles release excess oxygen which helps in complete and clean combustion of fuels there by reducing combustible gases and hydrocarbons which could have released into atmosphere and resulted in air pollution^8,9^.

Advancements in biodiesel technology offer sustainable and environmentally friendly solutions, as these resources are both inexhaustible and eco-friendly^10^. ShaikMasthanShareef et al.^11^ in their investigation used 20% diary waste scum methyl ester blend and evaluated engine performance and combustible parameters. The results revealed that the biodiesel blend outperformed conventional diesel in key performance and environmental aspects. Manjunath et al.^12^ used copper nano fluid with dairy scum biodiesel and evaluated performance combustion and emission behaviour. They found that 75 ppm nanoadditive fuel blend exhibited the better performance features compared to other nanoadditive fuel blends. M.Venkatachalam et al.^13^ examined the performance and emission features of neem biodiesel with MLE additive. They found that B20 + 1% MLE blend outperformed conventional diesel by achieving a 3.2% improvement in BTE and a 3.5% reduction in BSFC. Notably, it also contributed to lower emission of HC and CO by 17.5% and 12%, respectively. G. Ramakrishnan et al.^14^ investigated the influence of CNT on emission features of neem biodiesel. The results showed that incorporating CNT nanoparticles into this biodiesel led to a reduction in smoke emissions, NOx, HC, CO. Rathinam et al.^15^ focused on the impact of size of CeO_2_ on emission features of 4-s Diesel engine having neem biodiesel. They found a reduction in CO and HC emissions due to the addition of CeO_2_ nanoparticles. J, N., and Balasubramanian, D.^16^ conducted experiments to find the impact of CAO nanofluid on performance of diesel engine having ternary blends (Calophyllum, Neem biodiesel and diesel) fuel. They found an increment in BTE and decrease in emission because of the addition of nanofluids. Nayak, S. K., & Mishra, P. C^17^ investigated the impact of neem biodiesel and dimethyl carbonate additive on diesel engines under various operating conditions. The results revealed a reductionHC and CO emission with increase in the percentage of additives. MdModassir Khan et al.^18^ used various blends of neem biodiesel under varying load and maintaining a constant speed of 2000 rpm. Compared to conventional diesel, the quaternary blends exhibited reduced CO, UHC, and smoke emissions, attributed to enhanced combustion efficiency.

Advancements in nanotechnology have profoundly impacted the automotive industry, introducing a transformative concept known as “nano fuels” into the existing body of scholarly research^19^. Notably, nanoparticles synthesized through the aqueous precipitation method have been widely used in numerous scientific investigations, highlighting their potential for enhancing fuel performance and combustion efficiency^20^. The different nano fillers that can be used in biodiesel for performance improvements are graphene, CNT, alumina, copper oxide and combination of nano fillers. The nano fillers are dispersed in bio diesel blends such that even distribution of fillers is achieved. The nano fillers in small quantities in terms of PPM are generally dispersed and this fuel is used to test performance measures and emission characteristics of CI diesel engines^21^. Based on the previous studies, in this investigation, three different blends and three proportions of nano fillers are used.The bio fuel blends used are 90% diesel and 10% neem oil, 80% diesel and 20% of neem oil and 70%diesel and 30% neem oil with alumina nanoparticles in the concentration of 100 PPM, 150 PPM and 200 PPM respectively.

Response Surface Methodology (RSM) and machine learning algorithms serve as powerful tools for predicting the performance, emission, and combustion characteristics of neem biodiesel blended with alumina nanoparticles^22^. While RSM provides a systematic approach for optimizing key engine parameters^23^, machine learning enhances predictive accuracy by capturing complex nonlinear relationships, enabling more efficient fuel formulation and emission reduction strategies^24^. Table 1 presents a comprehensive summary of studies that have employed advance ML and RSM techniques for predicting and optimizing engine performance, emission, and combustion characteristics. These studies highlight the synergistic advantages of combining ML’s predictive accuracy with RSM’s optimization capabilities to enhance engine efficiency and reduce emissions.

**Table 1:** Summary of studies using ML and RSM methods for forecasting and improving engine responses.

Ref	Techniques	Parameters	Biodiesel	Remarks
^25^	ANN	Performance and emission parameters	Waste cooking oil	All correlation coefficients (r) over 0.99 and R^2^ values were beyond 0.98 for all variables
^26^	RSM with desirability	Engine load, biodiesel blend, and nanoparticle concentration	Mahua with CuO nanoparticle	RSM identified M20 with 60 ppm NP at 80% load as optimal (desirability: 0.9), with the model achieving a MAPE of 3%
^27^	RSM,Gradient Boosting (GBoost), Extreme Learning Machine (ELM)	BP, LCV, Blends	Moringaoleifera biodiesel with 1-hexanol and Zr_2_O_3_ nanoparticles	ELM achieved the highest accuracy (R^2^ = 0.9604), outperforming other models
^28^	ANN-ANFIS, RSM	Methanol molar ratio, catalyst amount, reaction time	Neem and castor	The ANFIS demonstrated superior performance in terms of R^2^, compared to the ANN in forecasting yield
^29^	ANN, RSM	Split injection parameter	Ammonia-biodiesel	ANN predicted all responses with R > 0.99, ensuring superior real-time reproducibility over RSM
^30^	ANFIS-NSGA-II and RSM	Engine load, biodiesel blend, and nanoparticle concentration	Leachate blends with nano-additives	Response produced by ANFIS-NSGA-II were more accurate and proficient in comparison to other models
^31^	ANFIS and RSM	EGT and all types of emissions	Nano diesel blended fuels	The test results and the ANFIS predictions show a significant correlation
^32^	DTR, ABR, ETR, GBR, LGBM, and XGBR	Engine load, compression ratio, blend ratio	Aloe vera biodiesel with MWCNT nanoparticles	The XGBR model demonstrates the most accurate predictions
^33^	Decision Tree and RSM	CR, injection time, injection pressure,	biogas-biodiesel blends	The decision tree-based models were robust with almost negligible mean squared errors
^34^	RSM	Load and compression ratios	Cassia fistula and Ricinuscommunis	RSM yielded correlation coefficients (R^2^) between 0.92 and 0.99 for all output parameters
^35^	AMT ML and multi-objective optimization RSM	Varying engine torque, speed	Sunflower oil	The AWOA demonstrates superior precision and a faster convergence rate compared to PSO

The novelty of this study lies in the pioneering use of nano-additives in neem oil-based biofuel at high dosage levels (100–200 ppm), a parameter that has not been explored by previous researchers. Furthermore, the identification of an optimal blend that minimizes CO and NOx emissions presents a significant advancement, offering a viable solution for improving engine performance and reducing emissions in compression ignition (CI) engines, particularly in heavy-duty vehicles within the global transportation sector.It begins by employing Response Surface Methodology (RSM), a statistical approach that systematically analyzes the complex interactions between multiple input variables—such as fuel blend composition, engine settings, and operational parameters—and output responses like fuel efficiency, emissions, and engine power. Through experimental design using RSMand Desirability based parametric optimization, the study effectively maps these interactions and identifies optimal conditions for biodiesel performance.

To further assess RSM’s effectiveness, its results are compared with advanced machine learning (ML) algorithms, including linear regression (LR), decision tree (DT), and random forest (RF). This comparison provides a thorough evaluation of the predictive accuracy and reliability of RSM versus modern data-driven models, highlighting their respective strengths and limitations in biodiesel optimization. By integrating these hybrid approaches, the study optimizes key performance factors such as combustion efficiency, emissions reduction, and fuel economy.This combined use of RSM and ML models not only fills existing research gaps but also presents a more comprehensive, efficient, and sustainable strategy for enhancing engine performance with biodiesel blends.

## Methodology

### Preparation of bio fuel blends

Initially the neem oil extracted from neem seeds is processed by transesterification process and three different blends with the following proportion are prepared as shown in Tables 2 and 3. The nano particles are suspended in these fuels with three different fuel systems viz., (1) diesel 90% with 10% bio fuel (2) diesel 80% with 20% bio fuel and (3) 70% diesel with 30% bio fuel and these fuels in-turn mixed with alumina particles in proportion of 100 PPM, 150 PPM and 200 PPM to prepare bio fuels for the proportion of bio fuels with nano particles by using ultrasonicator to ensure proper distribution of nano particulates. In blending the bio fuels with nano additives, the agitation time and frequency are important parameters and were set as per standards for a duration of 25–30 min at 49 kHz and were maintained to ensure proper dispersion of alumina particles. Table 2 shows the properties of alumina nanoparticles. The thermo physical properties of various bio diesel blends dispersed with nanoparticles are shown in Table 3.

**Table 2:** Properties of nano particles (alumina (Al_2_ O_3_)).

Particle size	< 50 nm
Purity	99.9%
Form	Powder
Colour	White
Surface area	12–18 m^2^/g
Density	3.9 g/cm^3^

**Table 3:** Thermo physical properties of biodiesel blends. Suspended with alumina nanoparticles.

Properties	ASTM standards	B10 (diesel90% + 10% bio fuel)			B20 (diesel80% + 20% bio fuel)			B30 (diesel70% + 30% bio fuel)		
		100 PPM	150 PPM	200 PPM	100 PPM	150 PPM	200 PPM	100 PPM	150 PPM	200 PPM
Viscosity (Cst) or (mm^2^/s)	ASTMD445	2.39	2.42	2.56	2.45	2.51	3.0	2.57	2.67	2.72
Density (Kg/m^3^)	D5002	820.1	822.35	824.65	825.9	827.24	829.76	831.4	834.12	836.97
Flash point (°C)	ASTM D92	85	83	83	84	79	78	85	83	84
Fire point (°C)	ASTM D92	91	93	92	91	90	89	91	90	91
Calorific value (KJ/Kg)	EN14314	41,195	41.298	41,563	41,156	41,510	41,678	41,234	40,998	41,198
Pour point (°C)	ASTMD99-12	3	2	1	3	2	1	4	3	2

### Experimental setup

**Fig. 1:**
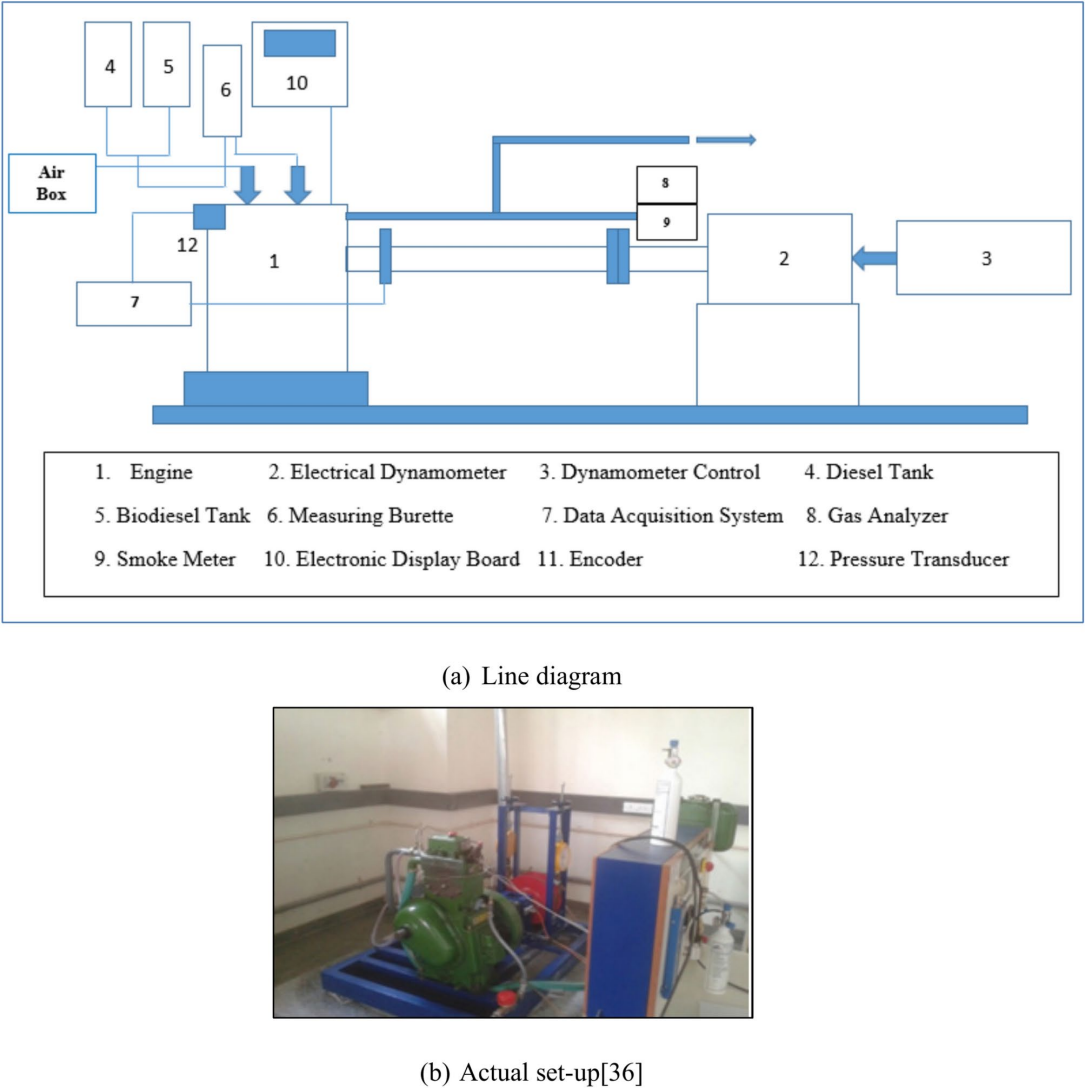
Experimental setup.

The experimental work has been carried out on single cylinder 4- stroke CI engine coupled with eddy current dynamometer and gas analyser as shown in Fig. 1.Three trails for each parameters /fuel blends are carried out and average values are considered for evaluation of performance measures and emission features. The engine started and run at rated speed and readings are taken after steady state is reached. The readings are recorded for lower and higher loading and medium loading conditions. Table 4 shows the specification of Engine Test rig and Table 5 shows specifications of the gas analyzer used for measurement of exhaust gases with its accuracy. The presence of exhaust gases like CO, Hydrocarbons and Nitrous oxide are measured using gas analyzer by vol% and PPM respectively.

**Table 4:** Specification of engine test rig.

Type	Kirloskar 1 cylinder 4 stroke water cooled engine
Bore 0 mm	87.50 mm
Stroke	110 mm
Combustion type	Compression ignition
Engine capacity	661 cm^3^
Power	3.5 Kw at 750 Rpm
Speed	759–2000 rpm

**Table 5:** Specifications of gas analyzer and smoke meter.

Gas analyzer model	AVL Digas 444	
Pollutant	Range	Accuracy
CO	0–10% vol	0.025% Vol
HC	0–21,000 PPM	+ 10 PPM
NO_x_	0–4999 PPM	+ 10 PPM
CO_2_	0–20% vol	0.5%vol
O_2_	0–22% vol	0.1% vol

### Machine learning (ML)

ML algorithms play a crucial role in biodiesel research by enhancing predictive accuracy and optimizing fuel performance. The complexity of biodiesel combustion, influenced by multiple interacting parameters such as blend composition, engine settings, and operating conditions, necessitates advanced data-driven approaches. ML models can efficiently analyze vast datasets, identify hidden patterns, and generate reliable predictions for fuel efficiency, emissions reduction, and engine performance. By integrating ML with conventional techniques like Response Surface Methodology^37^, researchers can develop more robust and adaptive optimization strategies, accelerating the development of sustainable and high-performance biodiesel fuels^38^.

### Linear regression

One of basic statistics methods is linear regression. It approximates the link between a dependent variable and one or more independent variables. Simple linear regression—one dependent and one independent variable—is the easiest version^39^. It treats variables as having a straight-forward connection. A straight line’s equation shows this link: *y* = *mx* + *c*, where *m* is the slope and *c* is the intercept. Linear regression seeks to identify the line of greatest fit. It reduces the total of the squared deviations from observed to expected values. One may grasp and use this approach easily. Still, it has some restrictions^40^. It takes linearity, which is not always the case. The outcomes can be much changed by outliers. Furthermore, challenging might be multicollinearity among independent variables. Linear regression is still somewhat often employed despite these disadvantages^41^. In disciplines including economics, finance, and the social sciences particularly it is quite popular. It forms a baseline model in machine learning. One can relate more complicated models against it. All things considered; linear regression is a quite effective yet basic instrument. It offers insightful analysis of the links among the variables^42^.

### Decision tree

A decision tree is a flexible tool available in machine learning. Its applicability consists in both categorizing tasks and in regression. Branching and nodes define the tree building. Every node represents a choice motivated by a feature. Branches convey the outcome of these decisions. The tree divides data into categories based on most critical criteria^43^. This process continues until a stopping requirement is reached. The result is a tree in which every leaf node represents a last choice or result. Decision trees are obviously visible and understandable. They handle numerical and categorical data as well. Their key advantage is their ability to capture non-linear relationships. Still, decision trees may overfit easily^44^. This happens as the tree develops too complex and picks noise in the data. One can help to remedy this by using different pruning techniques. By simplifying the tree, one can reduce overfitting. Moreover, constrained by decision trees are imbalanced datasets. The performance may seem to be slanted in the direction of the minority class^45^. Still, decision trees are very common in spite of these challenges. Built on these are more intricate ensemble methods such random forests and gradient boosting. In conclusion, decision trees are very handy tool. Many vocations provide clear, reasonable results.

### Random forest

One approach in ensemble learning is random forest. It uses many decision trees in order to enhance performance. Random forests are a potent ensemble technique. To provide strong and accurate forecasts, they integrate the powers of several decision trees. Every tree in the forest undergoes random subset of data training. Bootstrapping is the method used in creation of this subgroup^46^. Furthermore, taken into account at every tree split is a random collection of characteristics. This randomization promotes generalization and helps to lower overfit. The random forest produces, at last, the average prediction of every individual tree. For classifying chores, it’s the majority vote. Robust and good for many applications are random forests. They quickly manage high-dimensional areas and big datasets^47^. Their capacity to manage missing values is one main benefit. They also project feature prominence. Random forests, meanwhile, can be computationally costly. Training several trees calls additional resources. Random forests are quite popular in usage despite these shortcomings. From banking to healthcare, they are successful in many spheres. Many times, they provide the standard for other methods^48^.

### Model evaluation

#### R-squared (R^2^)

The coefficient of determination, or R-squared, gauges the share of the dependent variable variation that is predicted from the independent variables^49^. It runs from 0 to 1; 0 denotes no predictive ability and 1 denotes flawless prediction.

$${R}^{2} = 1 - \frac{\sum_{i=1}^{n}{({y}_{i}-\widehat{{y}_{i}})}^{2}}{\sum_{i=1}^{n}{({y}_{i}-\overline{{y }_{i}})}^{2}}$$

### Mean squared error (MSE)

MSE calculates the expected and actual values’ average squared difference. It shows the nearness of the forecasts to the actual numbers^49^. Reduced values show improved fit.

$$MSE = \frac{1}{n}\sum_{i=1}^{n}{({y}_{i}-\widehat{{y}_{i}})}^{2}$$

### Mean Absolute percentage Error (MAPE)

MAPE reports the prediction accuracy as a percentage. It gauges the average absolute percentage difference between actual and projected values^50^.Reduced values point to improved accuracy.

$$MAPE = \frac{1}{n}\sum_{i=1}^{n}\left|\frac{{y}_{i}-\widehat{{y}_{i}}}{{y}_{i}}\right| \times 100\text{\%}$$

Herein, ${y}_{i}$ is the measured value for ith observation, $\widehat{{y}_{i}}$ is the forecasted value for ith observation, n is the total observations being considered, $\overline{{y }_{i}}$ is the mean value of observations.

### Violin cum box plots for model comparison

In machine learning, violin cum box plots combine the elements of violin plots and box graphs to offer a complete view for model comparison. With the width indicating the density of data points at various values, violin plots display the distribution of data among several models. A box plot showing the median, quartiles, and possible outliers sits inside the violin plot. Plotting the performance metrics—such as accuracy, R^2^, MSE—for every ML model helps one to evaluate them. The violin cum box plot will expose the distribution and dispersion of these measures. Whereas the form of the violin plot emphasizes the whole distribution, the box plot inside shows central tendency and variability. This display lets one find models with consistent performance, spot anomalies, and compare core trends and spreads. It makes it simpler to pick the most solid and dependable model as it offers a more thorough comparison than conventional box graphs^51^.

## ML modelling

### Data analysis and correlation values

**Fig. 2:**
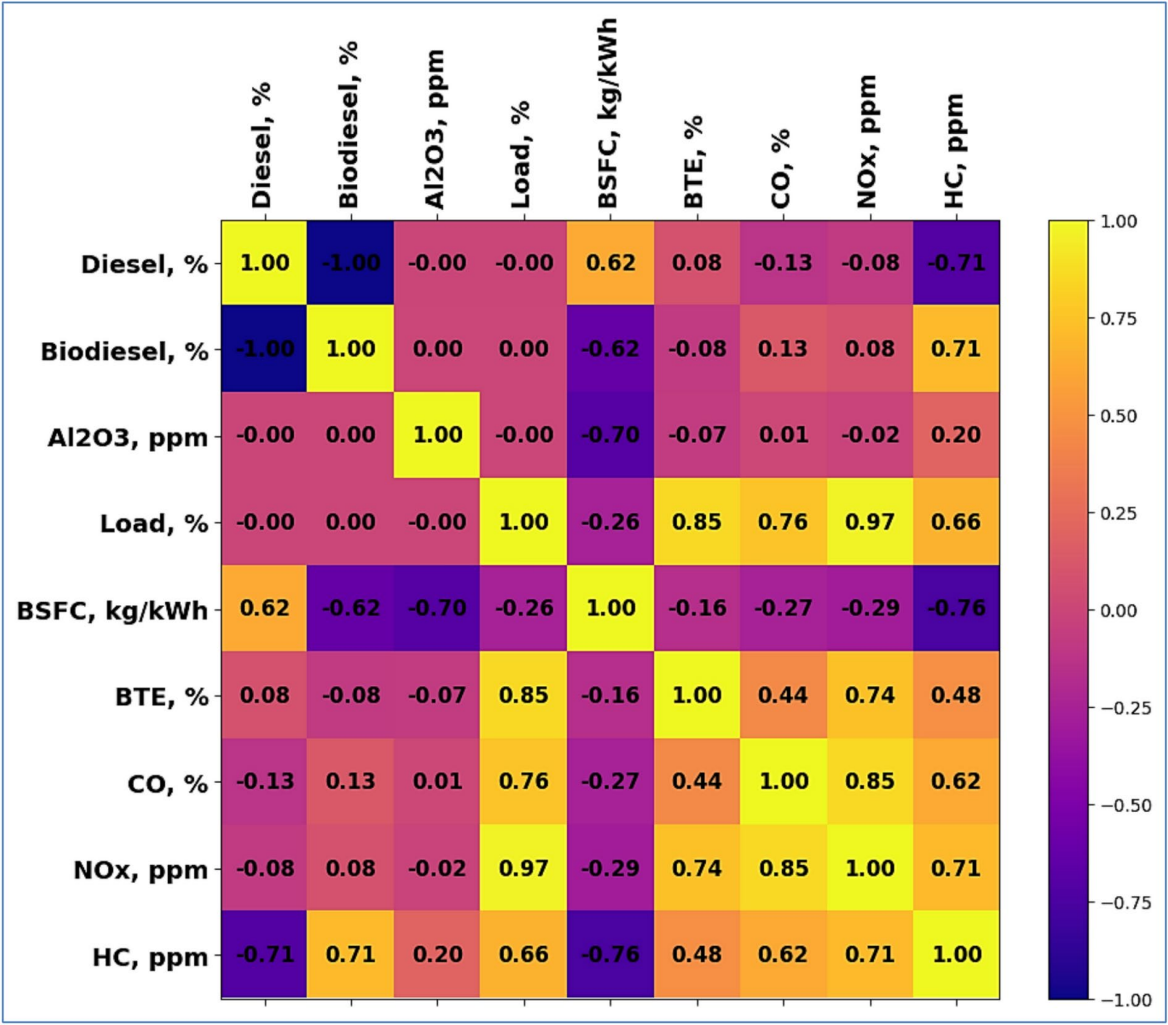
Correlation heat map.

The interactions among several elements influencing engine performance and emissions offer important new perspectives. The correlation heat map is depicted in Fig. 2. The correlation indices are listed in Table 6. Strong inverse correlation (− 1) indicates that when one climbs, the other falls; diesel and biodiesel percentages show this relation. Higher diesel percentage s show that diesel has a somewhat positive link with brake-specific fuel consumption (BSFC) (0.62). Al_2_O_3_ concentration is negatively linked with BSFC (− 0.7), meaning that increasing Al2O3 reduces fuel usage. Brake thermal efficiency (BTE) (0.85) has a substantial positive connection with the load; CO (0.76) and NOx (0.97) have quite high positive correlation as well. Higher loads, thus, both enhance efficiency and raise emissions. Higher fuel use lowers efficiency and raises emissions, according to BSFC’s negative association with BTE (− 0.16), CO (− 0.27), NOx (− 0.29), and HC (− 0.76). As efficiency rises, emissions also often tend to increase; BTE shows positive relationships with CO (0.44), NOx (0.74), and HC (0.48).

**Table 6:** Correlation values among parameters.

	Diesel, %	Biodiesel, %	Al_2_O_3_, ppm	Load, %	BSFC, kg/kWh	BTE, %	CO, %	NOx, ppm	HC, ppm
Diesel, %	1	− 1	0	0	0.62	0.08	− 0.13	− 0.08	− 0.71
Biodiesel, %	− 1	1	0	0	− 0.62	− 0.08	0.13	0.08	0.71
Al_2_O_3_, ppm	0	0	1	0	− 0.7	− 0.07	0.01	− 0.02	0.2
Load, %	0	0	0	1	− 0.26	0.85	0.76	0.97	0.66
BSFC, kg/kWh	0.62	− 0.62	− 0.7	− 0.26	1	− 0.16	− 0.27	− 0.29	− 0.76
BTE, %	0.08	− 0.08	− 0.07	0.85	− 0.16	1	0.44	0.74	0.48
CO, %	− 0.13	0.13	0.01	0.76	− 0.27	0.44	1	0.85	0.62
NOx, ppm	− 0.08	0.08	− 0.02	0.97	− 0.29	0.74	0.85	1	0.71
HC, ppm	− 0.71	0.71	0.2	0.66	− 0.76	0.48	0.62	0.71	1

Strongly positive correlation (0.85) between CO and NOx suggests they often rise simultaneously. With diesel (− 0.71) and BSFC (− 0.76), HC has negative correlations; with biodiesel (0.71), CO (0.62), and NOx (0.71), it exhibits positive relationships. Higher diesel use and fuel consumption hence lower HC emissions; higher biodiesel use and other contaminants raise HC emissions. These relationships underline the complicated connections among fuel types, engine load, and emissions, therefore directing the optimization of fuel mixes and running conditions for greater performance and reduced emissions.

### Model development and evaluation

The model evaluation highlights that Random Forest consistently outperforms Linear Regression and Decision Tree models across all parameters, demonstrating superior accuracy and generalization with minimal Lack of Fit. While Linear Regression struggles with complex relationships, as seen in high MSE and MAPE for various parameters. Decision Trees tend to overfit the training data, leading to less reliable test performance. Random Forest’s ability to balance bias and variance makes it the most robust model for this dataset, ensuring high R^2^ and low errors in both training and testing phases.

### BSFC models

**Fig. 3:**
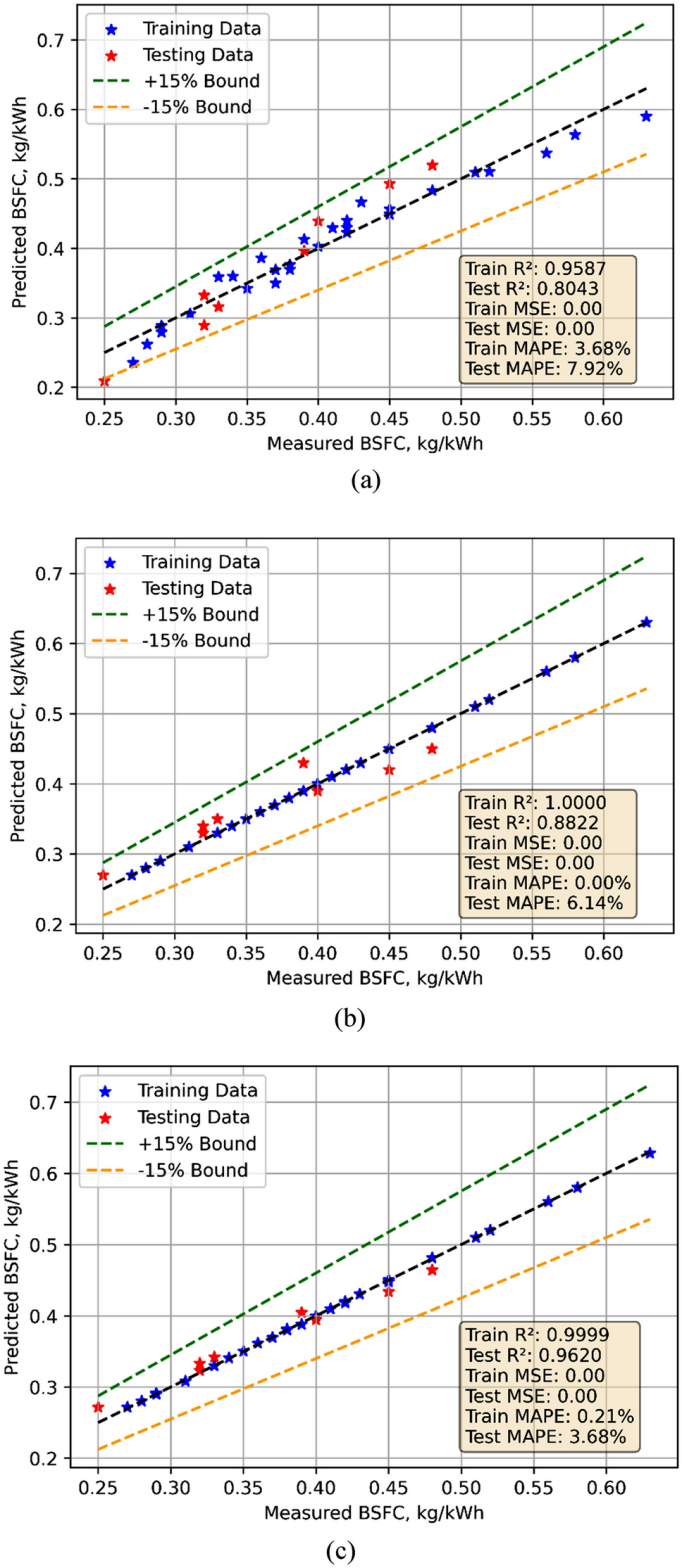
Model predicted versus actual BSFC values for (**a**) LR (**b**) DT (**c**) RF models.

**Fig. 4:**
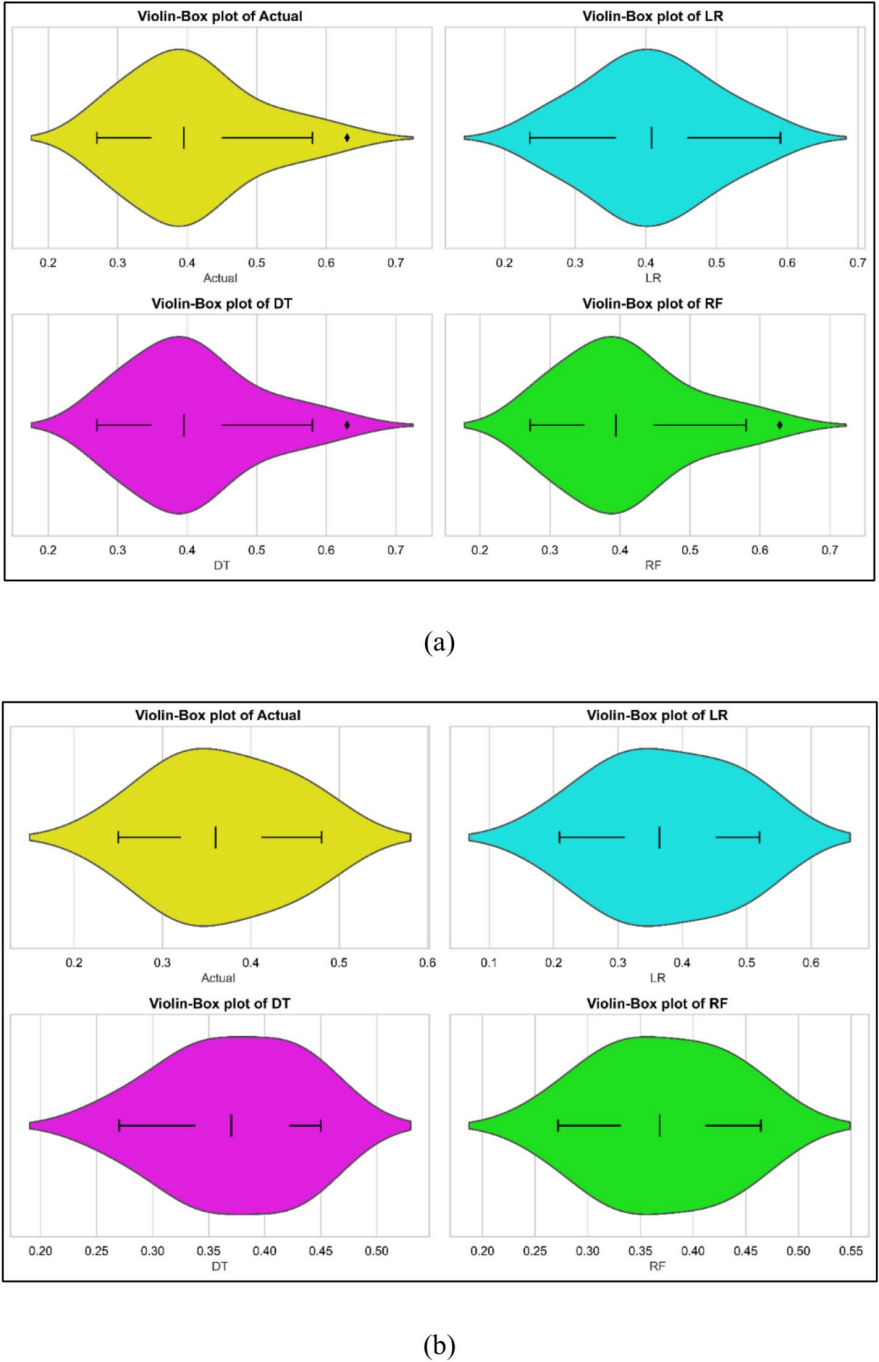
Violin cum box plots for BSFC models during (**a**) training (**b**) testing.

The Statistical models were developed using experimental data collected in testing. The 70% data was used for model training while remaining was used for model test. The models were used for prediction. The comparison between measured and predicted values are shown in Fig. 3a–c for all three models. All three models performed well. The models were evaluated using statistical methods. The results of statistical evaluations are listed in Table 7. With Train R^2^ of 0.9587 and Test R^2^ of 0.8043, LR’s Train MSE is 0.0003 and Test MSE is 0.0010, therefore showing a decent fit but more inaccuracy on test data. Training MAPE is 3.6825%; test MAPE is 7.9165%. Test R^2^ of 0.8822 and Test MAPE of 6.1386% imply that DT model displays ideal training fit—Train R^2^ of 1. With Train MSE almost zero, Test MSE of 0.002, Train R^2^ of 0.9999, and Test R^2^ of 0.9620 RF works best. Test MAPE is 3.6795%; Train MAPE is minimal—0.2148%. The violin cum box plots also corroborates the finding the statistical findings as depicted in Fig. 4a, b to show that RF model performed best.

**Table 7:** Lack of fit for the developed models.

Parameter	Model	Train MSE	Test MSE	Train R^2^	Test R^2^	Train MAPE	Test MAPE
BSFC	LR	0.0003	0.0010	0.9587	0.8043	3.6825	7.9165
	DT	0	0.0006	1	0.8822	0	6.1386
	RF	0	0.0002	0.9999	0.9620	0.2148	3.6795
BTE	LR	6.74	10.1520	0.8177	0.5290	7.0243	8.1527
	DT	0	5.9991	1	0.7217	0	6.8197
	RF	0	0.8798	1	0.9592	0.0033	2.7706
CO	LR	0.45	0.9108	0.5920	0.5489	107.71	114.28
	DT	0	0.0358	1	0.9823	0	9.8956
	RF	0	0.0351	1	0.9826	0.2071	9.3067
HC	LR	0.703	0.1040	0.9717	0.9972	1.302	0.5109
	DT	0	6	1	0.8389	0	4.1744
	RF	0	2.2642	1	0.9392	0.0026	2.2773
NOx	LR	781.5	342.0372	0.9425	0.9760	14.37	8.8571
	DT	0	349.3750	1	0.9754	0	6.5480
	RF	0	346.5180	1	0.9756	0.0006	7.2056

### BTE models

**Fig. 5:**
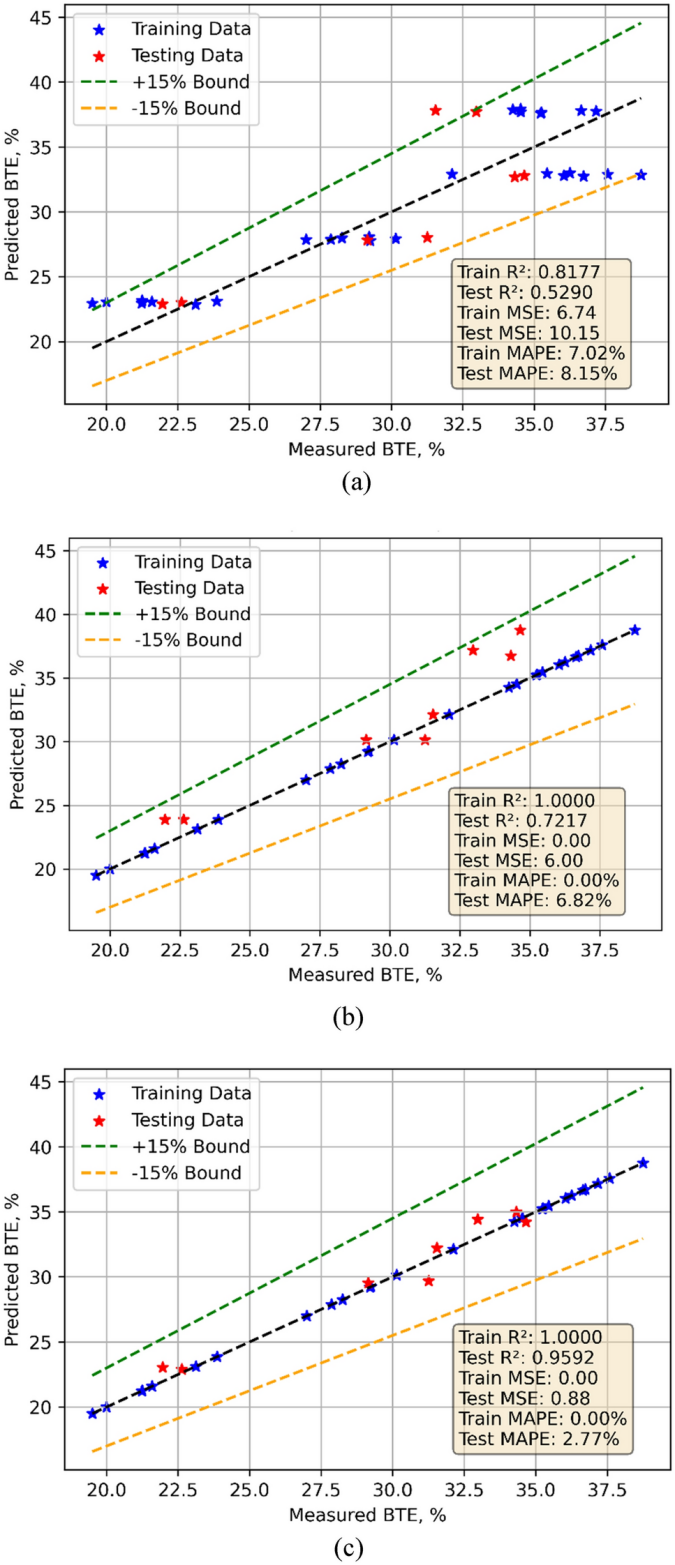
Model predicted versus actual BTE values for (**a**) LR (**b**) DT (**c**) RF models.

**Fig. 6:**
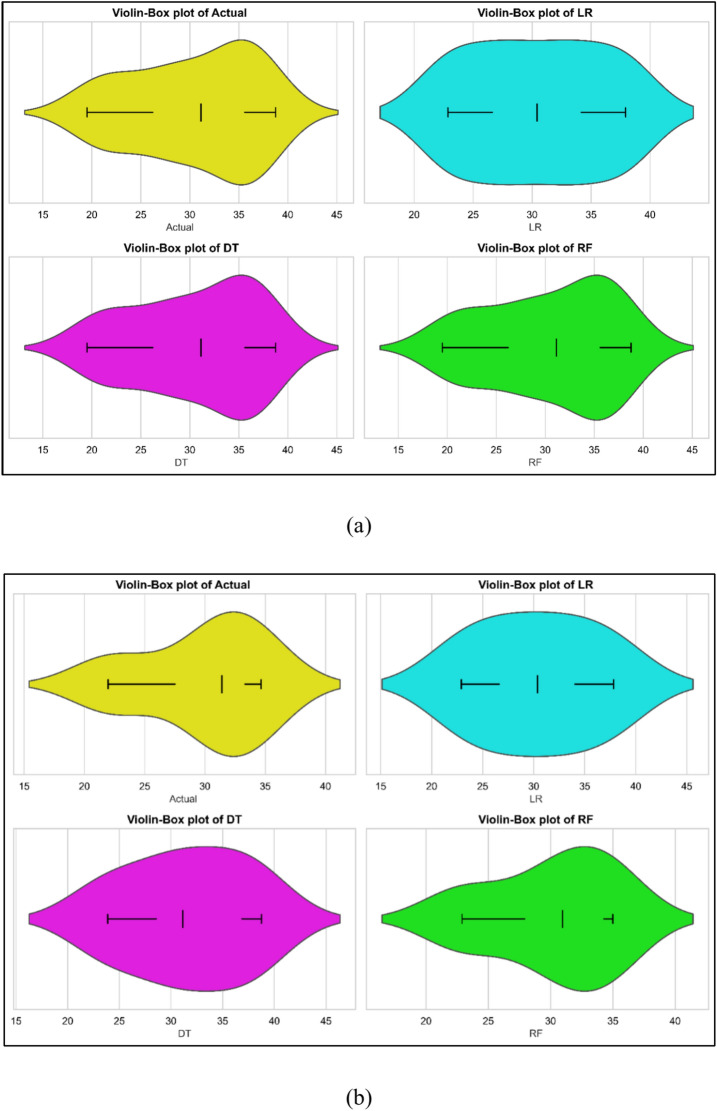
Violin cum box plots for BTE models during (**a**) training (**b**) testing.

Experimental data gathered in testing was employed for the development of BTE models. Model training took use of the 70% data; model testing made use of the remaining data. Predicts made using the models as Fig. 5a–c shows for all three models the contrast between observed and expected values. All three models did well. Statistical techniques were used in evaluation of the models. Table 7 lists the findings of statistical assessments. Three models—linear regression (LR), decision tree (DT), and random forest (RF)—have their BTE model outcomes compared with one another. With a Train MSE of 6.74 and a Test MSE of 10.1520 LR exhibits a modest performance suggesting possible overfitting. Reflecting smaller predictive power on the test set, the Train R^2^ is 0.8177 and the Test R^2^ is 0.5290. The test MAPE is 8.1527%; the train MAPE is 7.0243%. With a Test MSE of 5.9991 and a Test R^2^ of 0.7217, DT shows improved generalization but perfect training fit—Train MSE = 0, Train R^2^ = 1. With almost flawless training (Train MSE = 0, Train R^2^ = 1) and outstanding test performance (Test MSE = 0.8498, Test R^2^ = 0.959 RF beats both. Its lowest MAPE values point to strong accuracy and prediction resilience^41^. The violin cum box plots further supports the statistical data displayed in Fig. 6a, b, demonstrating that the RF model performed superior to other approaches.

### CO emission models

**Fig. 7:**
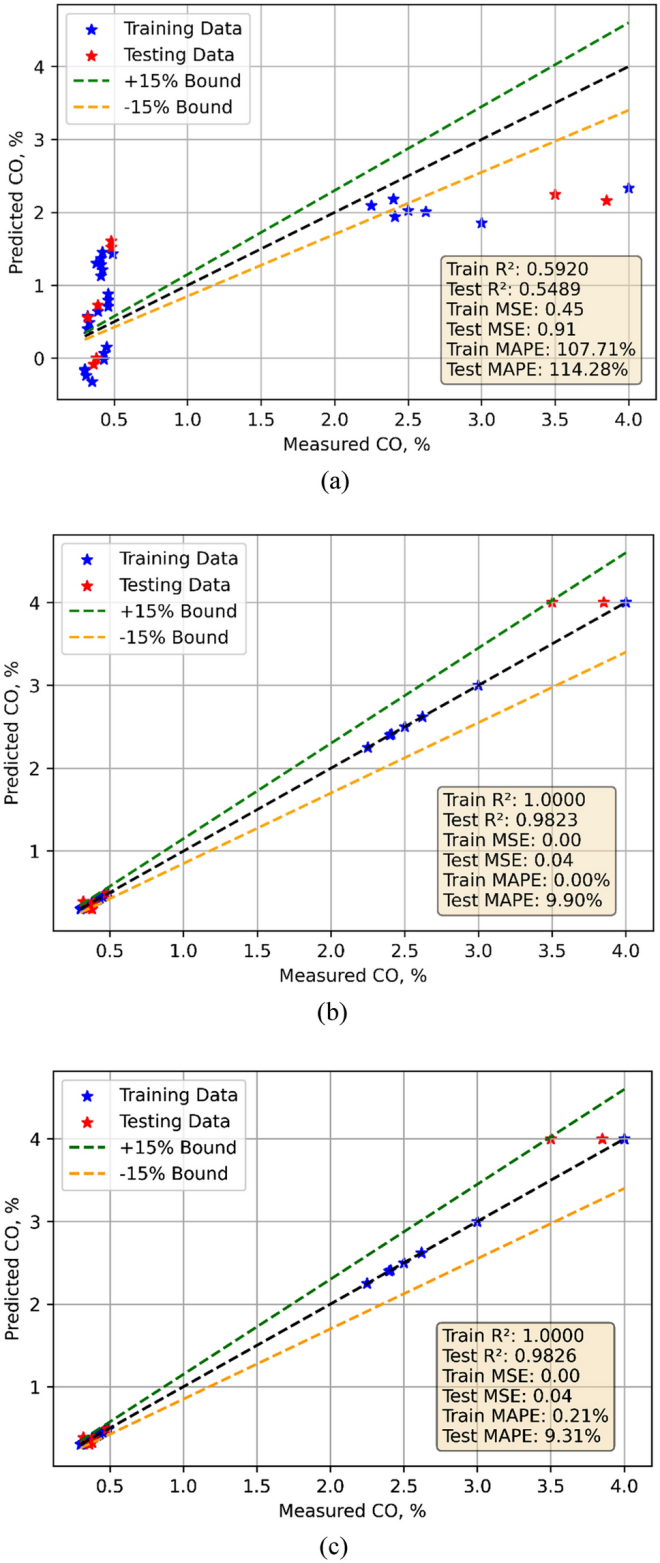
Model predicted versus actual CO emission values for (**a**) LR (**b**) DT (**c**) RF models.

**Fig. 8:**
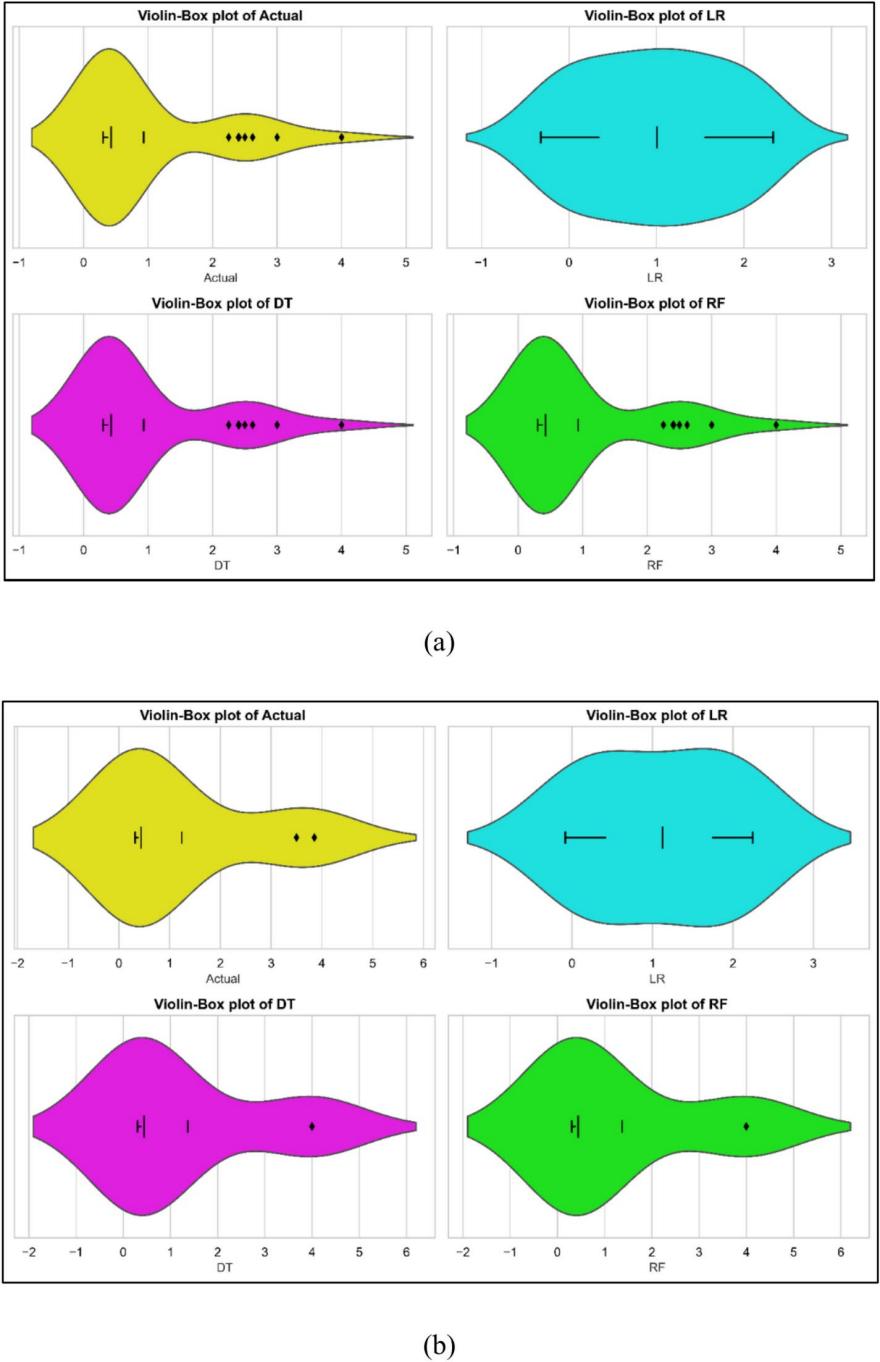
Violin cum box plots for CO emission models during (**a**) training (**b**) testing.

Development of the CO emission models drew on experimental data gathered during testing. Model training made use of the 70% data; model testing made use the remaining data. One could forecast using the models. Figure 7a–c displays for all three models the comparison between observed and projected values. Every three models performed rather well. Statistical approaches were used for evaluation of the models. Table 7 provides the statistical evaluation results. The performance criteria of several models for CO emissions expose varied strengths. With a Train MSE of 0.45 and a Test MSE of 0.9108 Linear Regression (LR) shows very modest performance. With a Train R^2^ of 0.5920 and a Test R^2^ of 0.5489 it shows a modest fit. The strong prediction errors shown by the high Train MAPE of 107.71% and Test MAPE of 114.28% point By comparison, models showing better performance include Random Forest (RF) and Decision Tree (DT). With a Train MSE of 0, both DT and RF indicate ideal training fit. At 0.0358 for DT and 0.0351 for RF, their Test MSEs are rather low. With Test R^2^ values of 0.9823 and 0.9826, respectively, the Train R^2^ for both models equals 1, indicating perfect training fit. While RF has somewhat better Test MAPE of 9.3067%, DT has a Test MAPE of 9.8956%. This indicates RF’s much stronger predictive accuracy. The violin cum box graphs show that the RF model performed better than other methods, therefore supporting the statistical data shown in Fig. 8a, b.

### NOx emission model

**Fig. 9:**
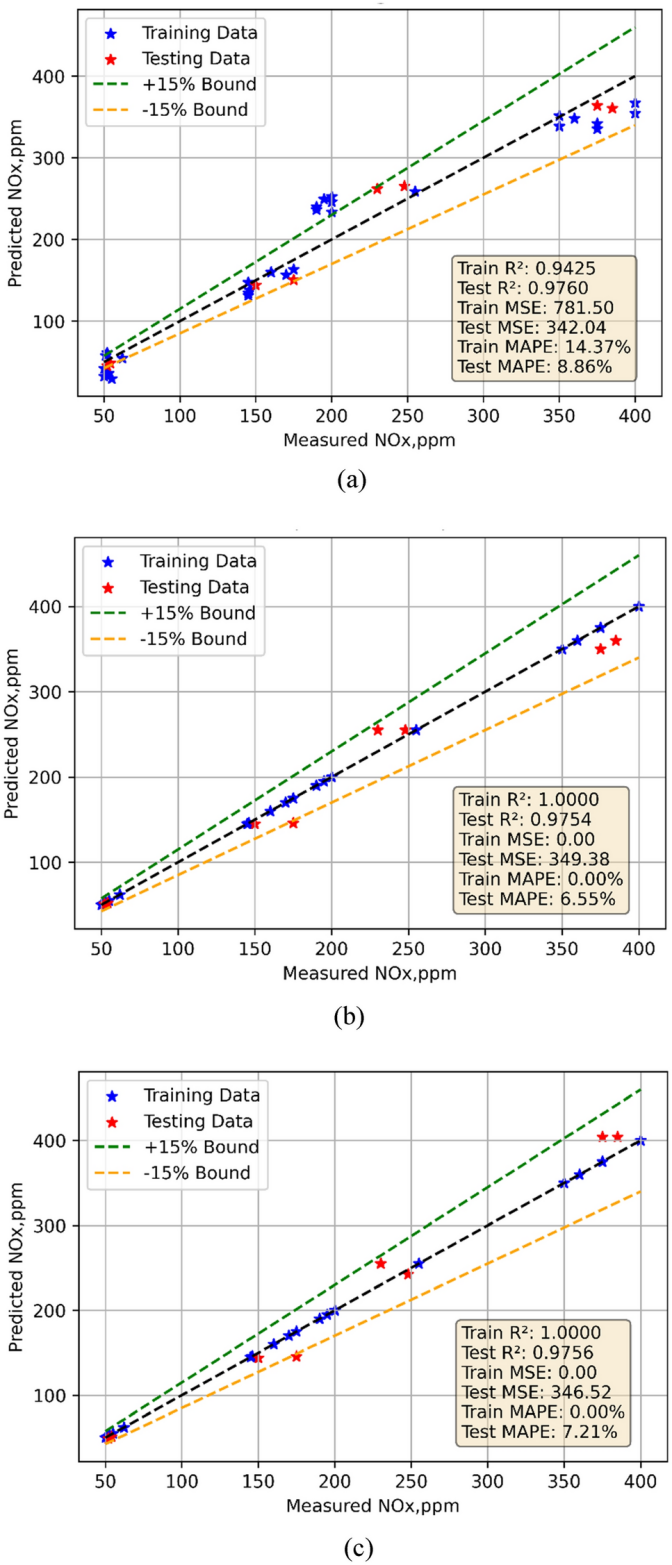
Model predicted versus actual NOx emission values for (**a**) LR (**b**) DT (**c**) RF models.

**Fig. 10:**
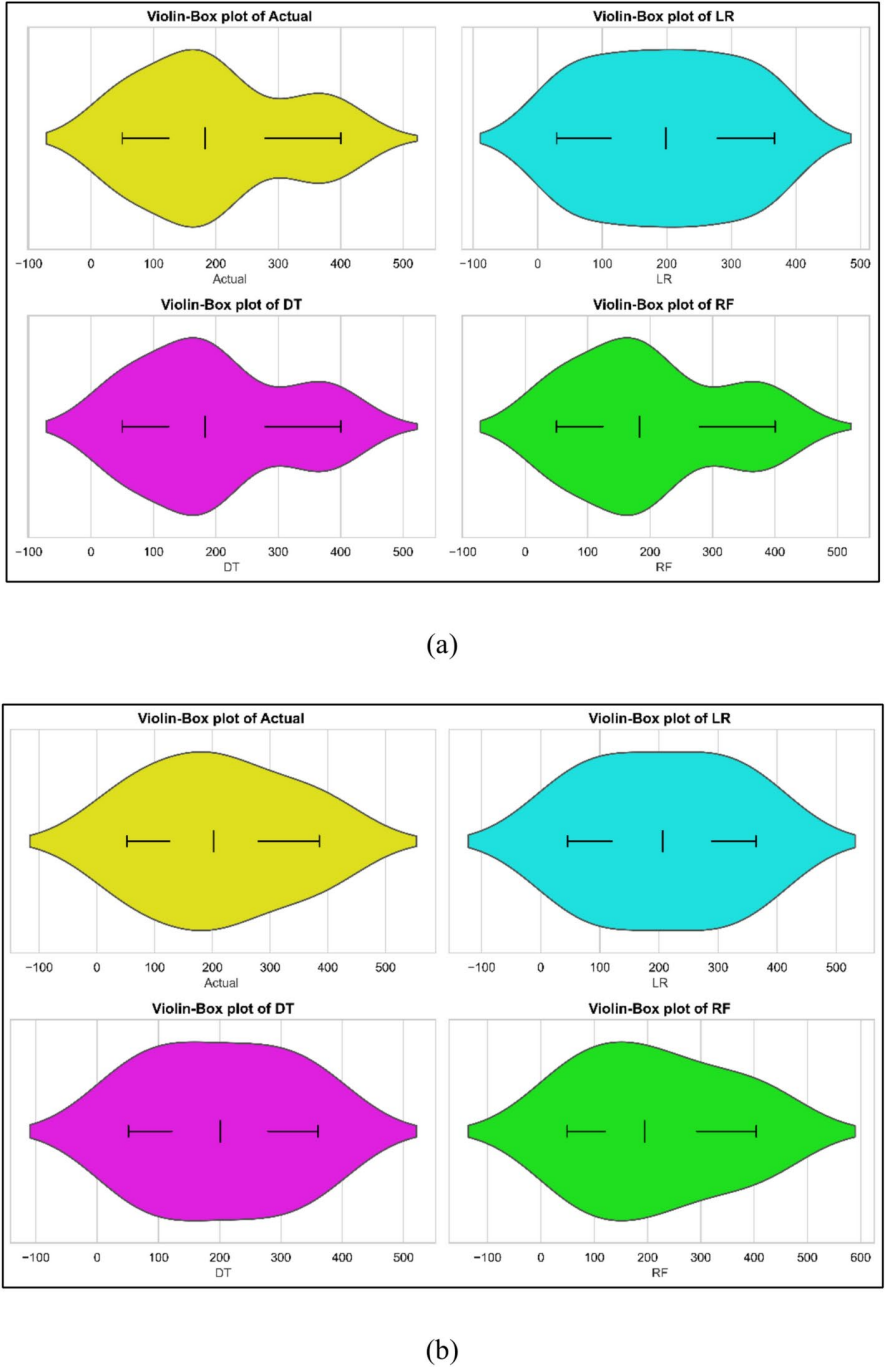
Violin cum box plots for NOx emission models during (**a**) training (**b**) testing.

The NOx emission models developed using experimental data acquired during testing. Using the 70% of the data, model training; using the remaining data, model testing. One might make forecasts with the models. Figures 9a–c show for each of the three models the observed against anticipated values comparison. Every three models showed fairly good performance. The models were evaluated using statistical methods. Table 7 offers the findings of statistical analysis. The models of NOx emission show somewhat different performance criteria. With a train MSE of 0.703 and a test MSE of 0.1040, the Linear Regression (LR) model exhibits excellent performance showing outstanding prediction accuracy. The model explains most of the variation in both sets according to the train R^2^ of 0.9717 and test R^2^ of 0.9972. Especially in testing (0.5109), the low MAPE values point to little prediction error. Showing overfitting, the Decision Tree (DT) model exhibits zero train MSE and R^2^ of 1. On fresh data, the test MSE of 6 and test R^2^ of 0.8389 point to inferior performance. Its low generalizability is especially confirmed by the high test MAPE (4.1744). With a test MSE of 2.2642 and test R^2^ of 0.9392, the Random Forest (RF) model balances generalizability and accuracy very well. With very low train and test MAPE values—0.0026 and 2.2773, respectively—it also indicates consistent predictions with low error. For NOx emissions prediction, the LR and RF models do generally better. The violin cum box graphs show that the RF model performed better than other methods, therefore supporting the statistical data shown in Fig. 10a, b.

### HC models

**Fig. 11:**
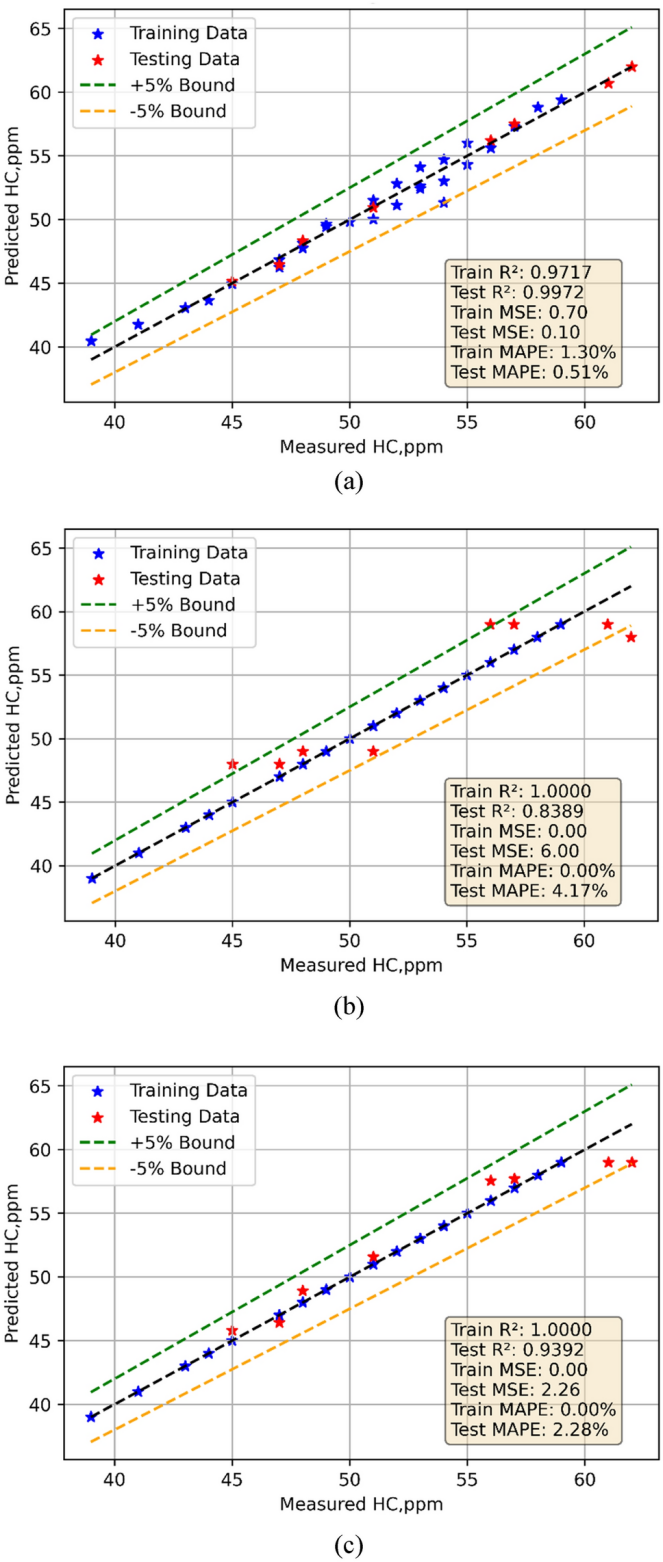
Model predicted versus actual HC emission values for (**a**) LR (**b**) DT (**c**) RF models.

**Fig. 12:**
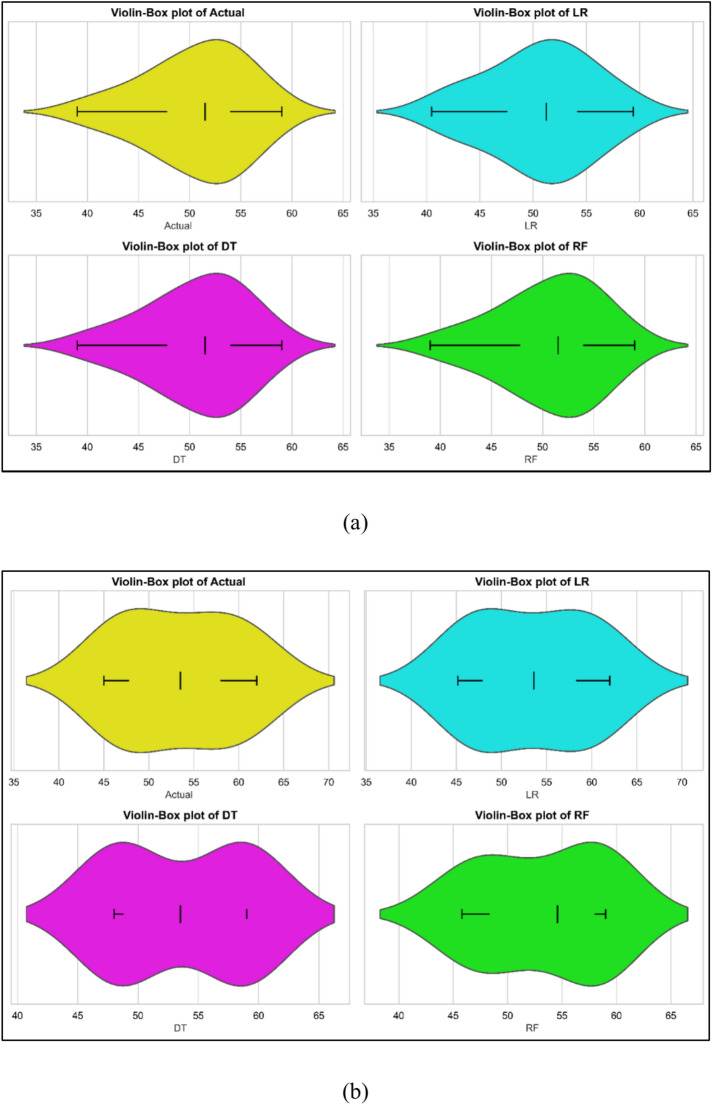
Violin cum box plots for HC emission models during (**a**) training (**b**) testing.

In the present study, the ML models were developed utilizing experimental data obtained during testing. Model training uses the 70% of the data; model testing uses the remaining data. One might use the models to create projections. Figures 11a–c depict for every one of the three models the observed against expected values comparison. Every three models displayed rather decent performance. Statistical techniques were used for evaluation of the models. Table 7 presents statistically analytical results.The performance measures of three models—Linear Regression (LR), Decision Tree (DT), and Random Forest (RF)—for HC emissions are shown in the table. Whereas the test MSE is lower at 342.0372, suggesting superior performance on unknown data, the train MSE for LR is 781.5, indicating a little mistake during training. Strong model fit is shown by the train R^2^ of 0.9425 and the test R^2^ of 0.9760. Showing acceptable prediction accuracy, the train MAPE is 14.37%; the test MAPE is 8.8571%. With zero train MSE, the DT model indicates ideal training performance; its test MSE, 349.3750, is somewhat greater. There is overfitting shown by the test R^2^ of 0.9754 when the train R^2^ is 1. With a 0% train MAPE and a 6.5480% test MAPE demonstrating great prediction accuracy, furthermore displaying zero train MSE and a test MSE of346.5180 is RF. Once more highlighting overfitting, the train R^2^ is 1 and the test R^2^ is 0.9756. The test MAPE is 7.2056%, showing accurate predictions with little error; the train MAPE is virtually nil. The violin cum box graphs show that the RF model performed better than other methods, therefore supporting the statistical data shown in Fig. 12a, b.

### Discussion on lack of fit (LoF) for different models

The detailed discussion of the lack of fit for different models is tabulated in Table 7. The table gives an overview of the training and testing mean squared errors (MSE), training and testing coefficient of determination (R^2^), training and testing Mean Absolute Percentage Error (MAPE) for different set of parameters.

BSFC: Linear Regression (LR) shows a significant lack of fit with a higher Test MSE (0.0010) compared to Train MSE (0.0003) and a lower Test R^2^ (0.8043). Decision Tree (DT) reduces LoF but suffers from overfitting with perfect Train R^2^. Random Forest (RF) performs the best, with minimal LoF indicated by consistent low Test MSE (0.0002) and high Test R^2^ (0.9620).

BTE: LR exhibits considerable LoF, with higher Test MSE (10.15) and low Test R^2^ (0.5290). DT improves Test performance but remains prone to overfitting. RF delivers the best results with near-perfect R^2^ and low Test MAPE (2.77%), indicating minimal LoF.

CO: LR has a high LoF, with a large gap in Train/Test MSE and extremely high Test MAPE (114.28%). DT and RF achieve low Test MSE and high Test R^2^ (0.9823 and 0.9826, respectively), showing minimal LoF and good generalization.

HC: LR performs well with low LoF, evidenced by a high Test R^2^ (0.9972) and low Test MAPE (0.51%). DT shows signs of overfitting and higher LoF with Test MSE (6). RF balances generalization and accuracy with minimal LoF.

NOx: LR shows reasonable generalization with lower Test MSE (342.0372) and high Test R^2^ (0.9760). DT and RF achieve similar performance, with RF showing slightly better metrics, including lower Test MAPE (7.21%).

In summary Random Forest consistently minimizes Lack of Fit across all parameters, delivering the best balance between accuracy and generalization. In contrast, Linear Regression often struggles, particularly with complex relationships. Decision Tree models frequently overfit but still perform well in certain scenarios.

## Results and discussion on performance and emission features

### Brake thermal efficiency

The significant factor that estimates the engine’s performance is defined as the amount of brake power developed by the engine relative to the calorific values and the amount of the mass of the fuel. It is technically expressed by the equation referred from^52^.

1 $$B_{{TE}} = \frac{{Brake\;power}}{{mass\;of\;fuel \times calorific\;value}}$$

The highest BTE is achieved for the blends which have the highest calorific values. The release of chemical energy from these fuels tends to accelerate the piston rapidly to achieve brake power.

**Fig. 13:**
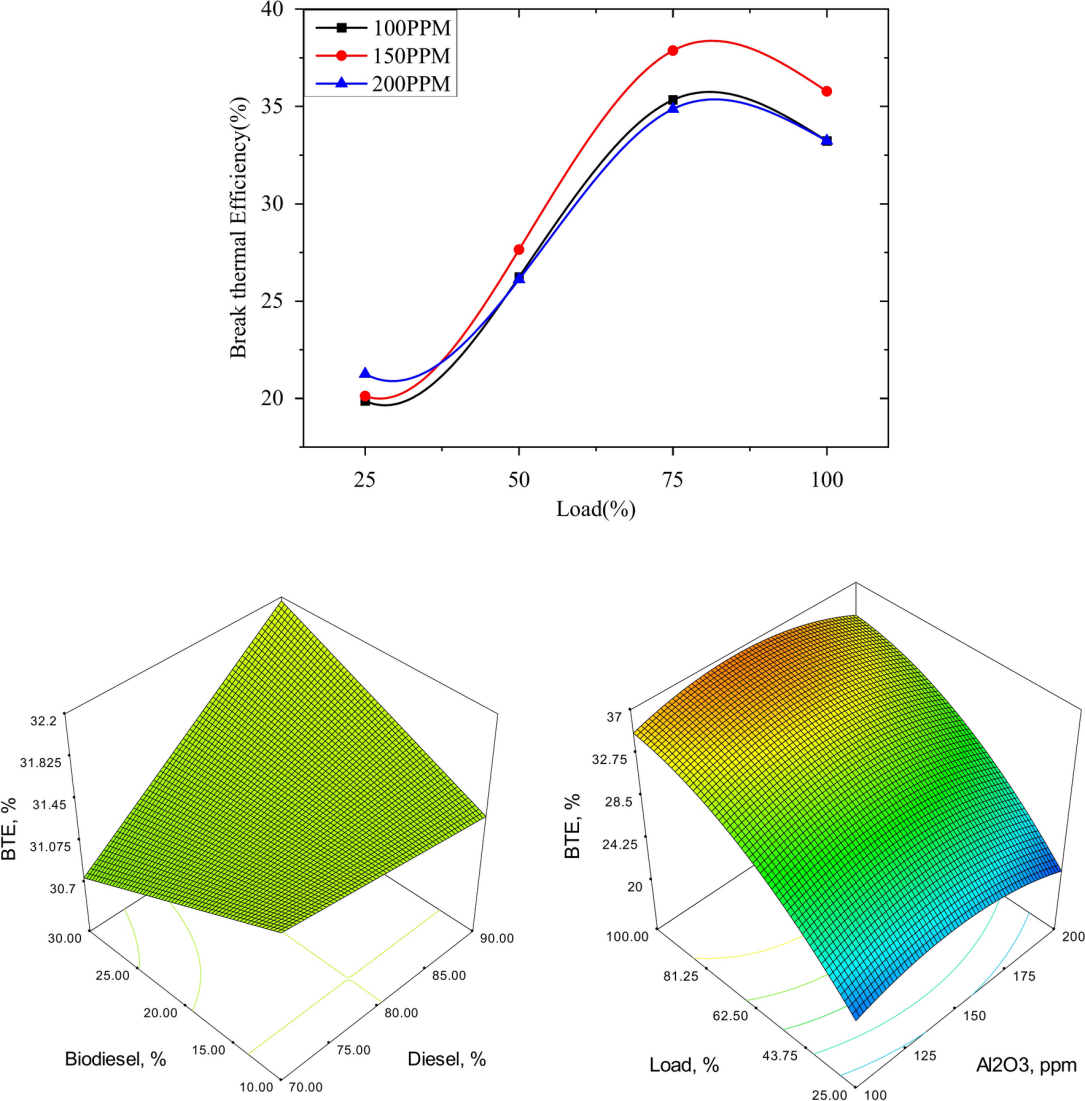
Variation of brake thermal efficiency versus load for 90% diesel and 10% bio fuel with nano particles.

**Fig. 14:**
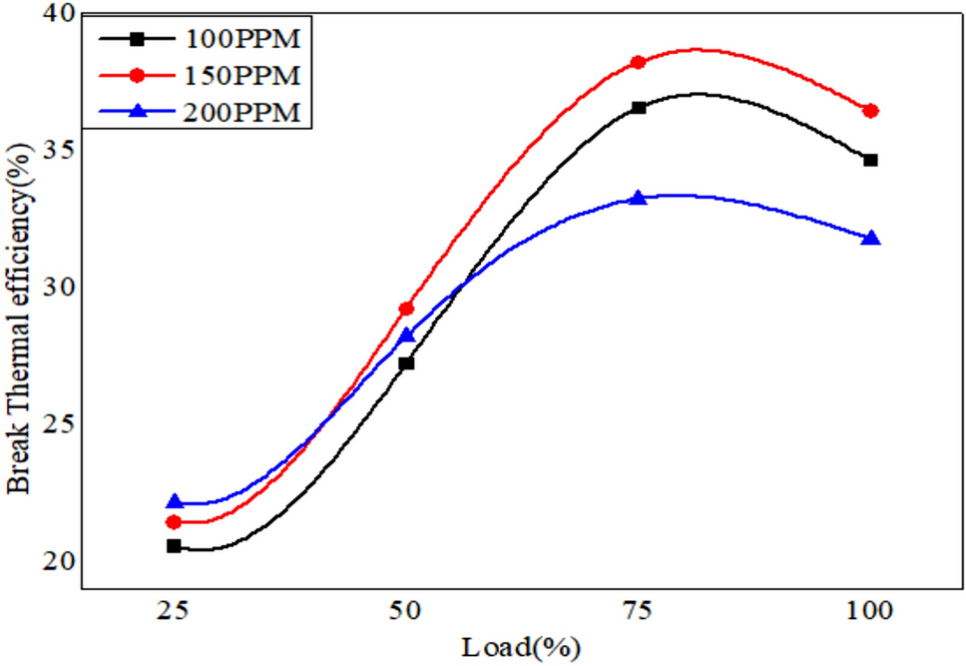
Variation of brake thermal efficiency versus load for 80% diesel and 20% bio fuel with nano particles.

**Fig. 15:**
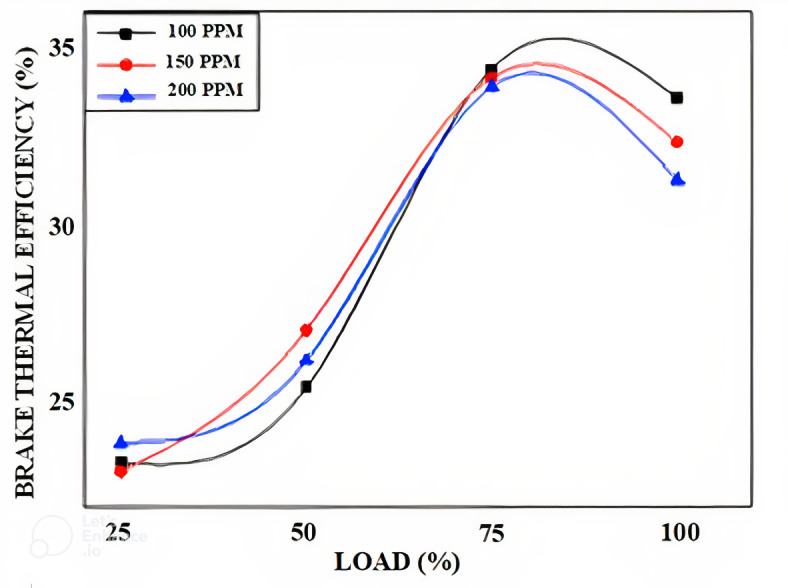
Variation of brake thermal efficiency versus load for 70% diesel and 30% bio fuel with nano particles.

The variation of brake thermal efficiency versus Load for different blends dispersed with different proportions if the nano alumina particles is as shown from Figs. 13, 14 and 15 with different proportion of nano particles. In our experimental work we have considered three different blends as 90% diesel blends with 10% bio neem oil which in turn is dispersed with alumina nano particles in 100 PPM.150 PPM and 200 PPM.The variation of break thermal efficiency for 25% 50%, 75% and Full load are evaluated and plotted. The results show that the neem oil blend with 20% bio fuel fairly gives good results compared to other blends, similarly nano particles dispersion with 150 PPM shows better results as these nano particles acts as catalyst to speed up combustion reaction and release oxygen which results in the complete combustion thereby improving thermal efficiency of the engine^53^.

The plots reveal that thermal efficiency increases with increase in load and will be maximum at ¾ load or 75% and it reduces further at full load capacity of engine, further the 80% diesel and 20% of bio fuel with 150 PPM nano particles shows better results when compared to 100 PPM and 200 PPM dispersion of nano particles for all the combinations of bio fuels^54^.

### Exhaust gas temperature (EGT)

A straightforward method to diminish NOx emissions in a diesel engine is by delayed fuel injection into the combustion chamber. This technology is efficacious but elevates fuel consumption by 10–20%, hence requiring the use of more efficient NOx reduction methods such as recirculation of exhaust gases (EGR). Re-circulating a portion of the exhaust gas mitigates NOx emissions; nonetheless, significant particulate emissions occur at elevated loads, indicating a trade-off between NOx and smoke release^55^. To optimize this trade-off, a particle trap may be employed to diminish the quantity of unburned nanoparticles in EGR, hence also reducing particulate emissions.

**Fig. 16:**
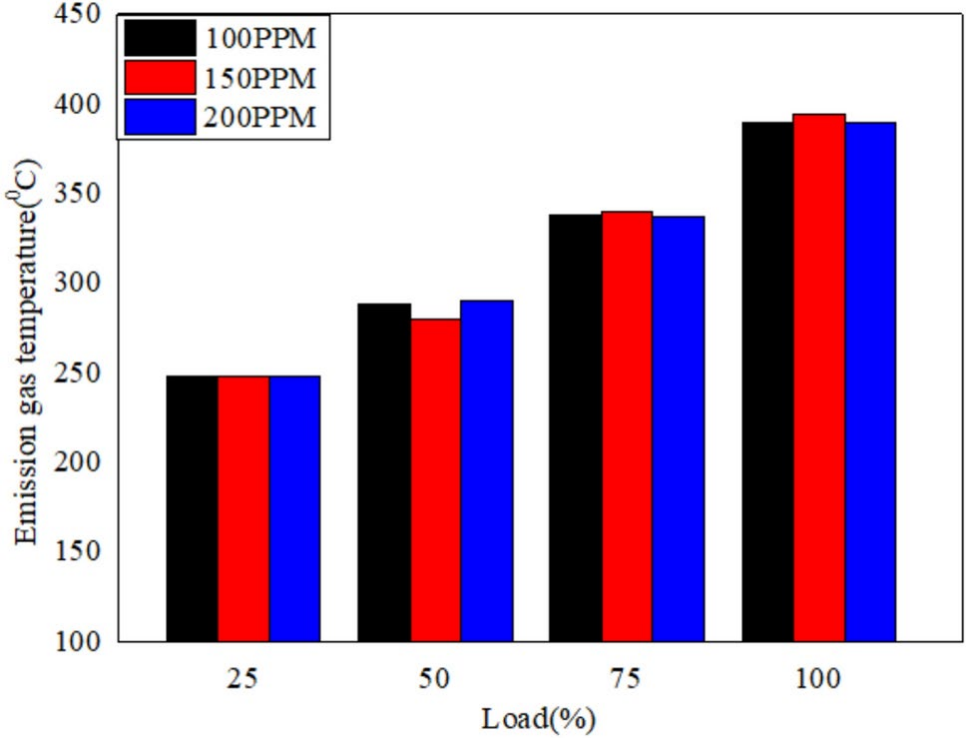
Variation of exhaust gas temperature versus load for different blends of biodiesel blend with 90% diesel and 10% biofuel with nano particles.

**Fig. 17:**
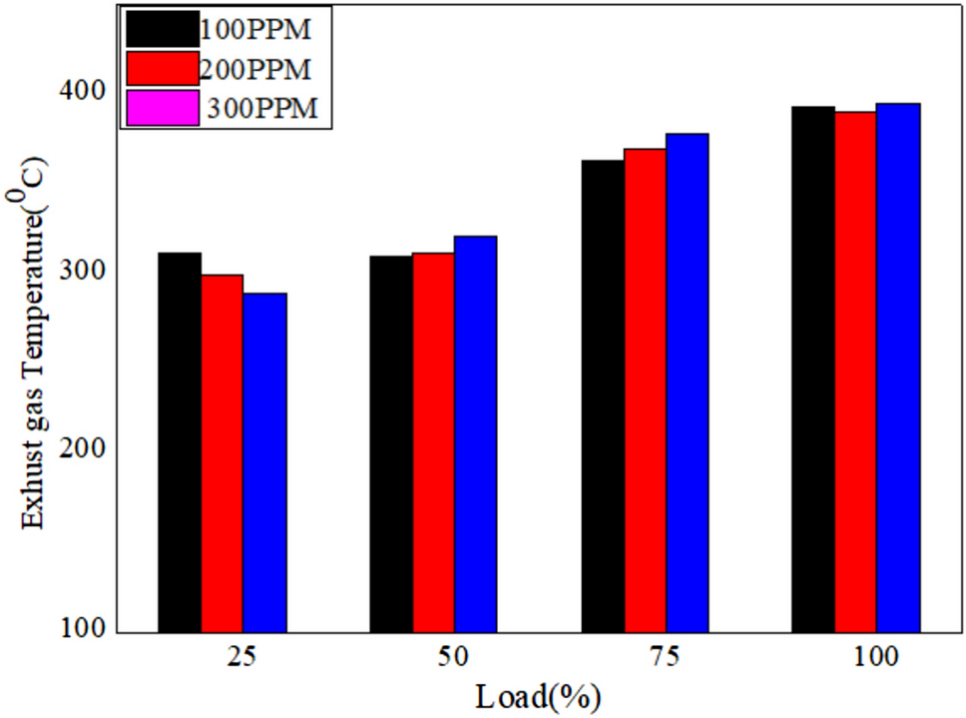
Variation of exhaust gas temperature versus load for different blends of biodiesel blend with 80% diesel and 20% bio `fuel with nano particles.

**Fig. 18:**
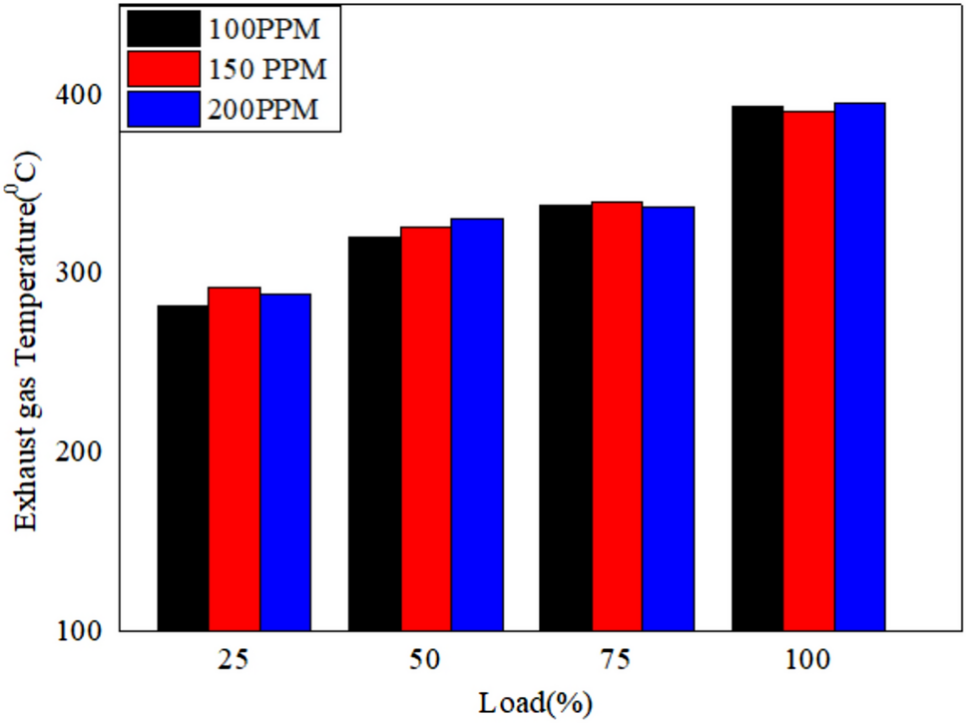
Variation of exhaust gas temperature versus load for different blends of biodiesel blend with 70% diesel and 30% biofuel with nano particles.

The EGT for various loads for different blends of bio fuels with nano particles dispersed in it is shown in Figs. 16, 17, and 18. The results reveal that the exhaust gas temperature increases with the load (%). Exhaust gas temperature for 150 PPM dispersion of nano particles improves the combustion characteristics^56^.

### Emission characterises of bio diesel blends dispersed with nano particles

**Fig. 19:**
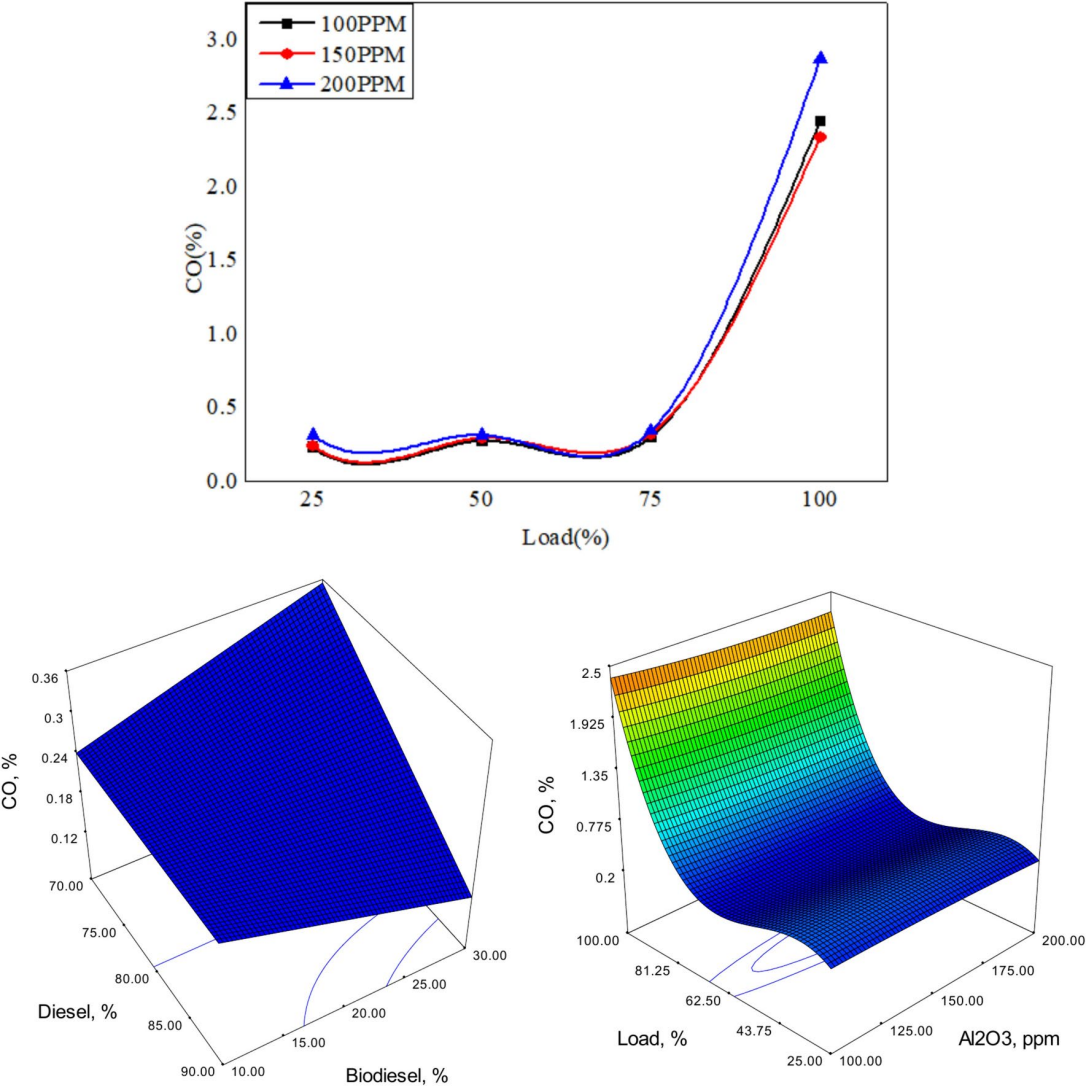
Variation of CO versus load for different blends of biodiesel blend with 90% diesel and 10% bio fuel with nano particles.

The emission characteristics is analyzed using gas analyzer as shown in Fig. 19. The combustion gases such as CO unburnt hydrocarbons and NO_x_ were analyzed.The variation of exhaust gases in vol% and PPM are plotted as shown below.

**Fig. 20:**
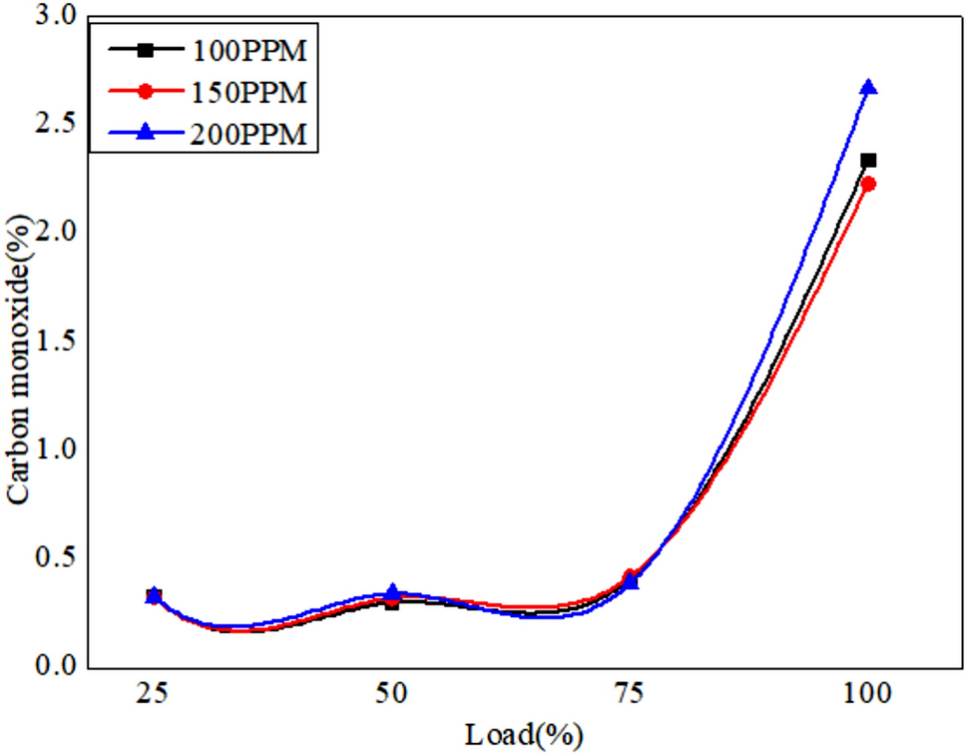
Variation of CO versus load for different blends of biodiesel blend with 80% diesel and 20% bio fuel with nano particles.

**Fig. 21:**
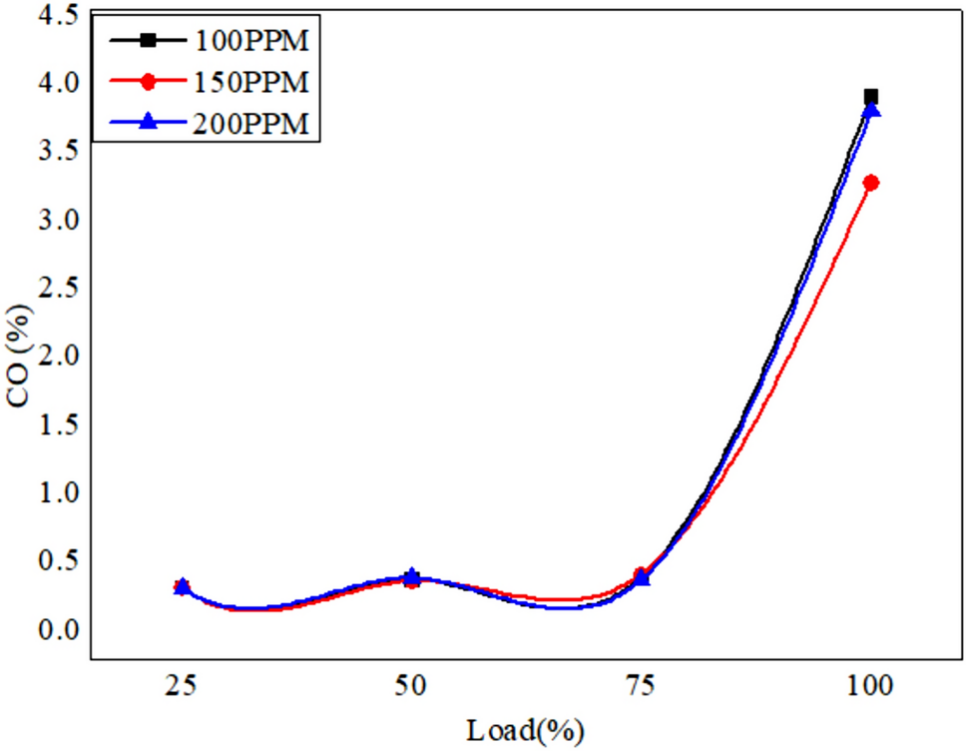
Variation of CO versus load for different blends of biodiesel blend with 70% diesel and 30% bio fuel with nano particles.

Figures 19, 20 and 21 depict the CO emissions in the CI engine for different loading conditions. The CO emission is almost the same for lower loading however at full load the CO emission is more as more fuel is consumed, and also due to the lag in ignition time^57^. However, the addition of nanoparticles ensures complete combustion of fuel due to the release of oxygen. Biofuel with 150 PPM shows better results compared to other fuel systems.

**Fig. 22:**
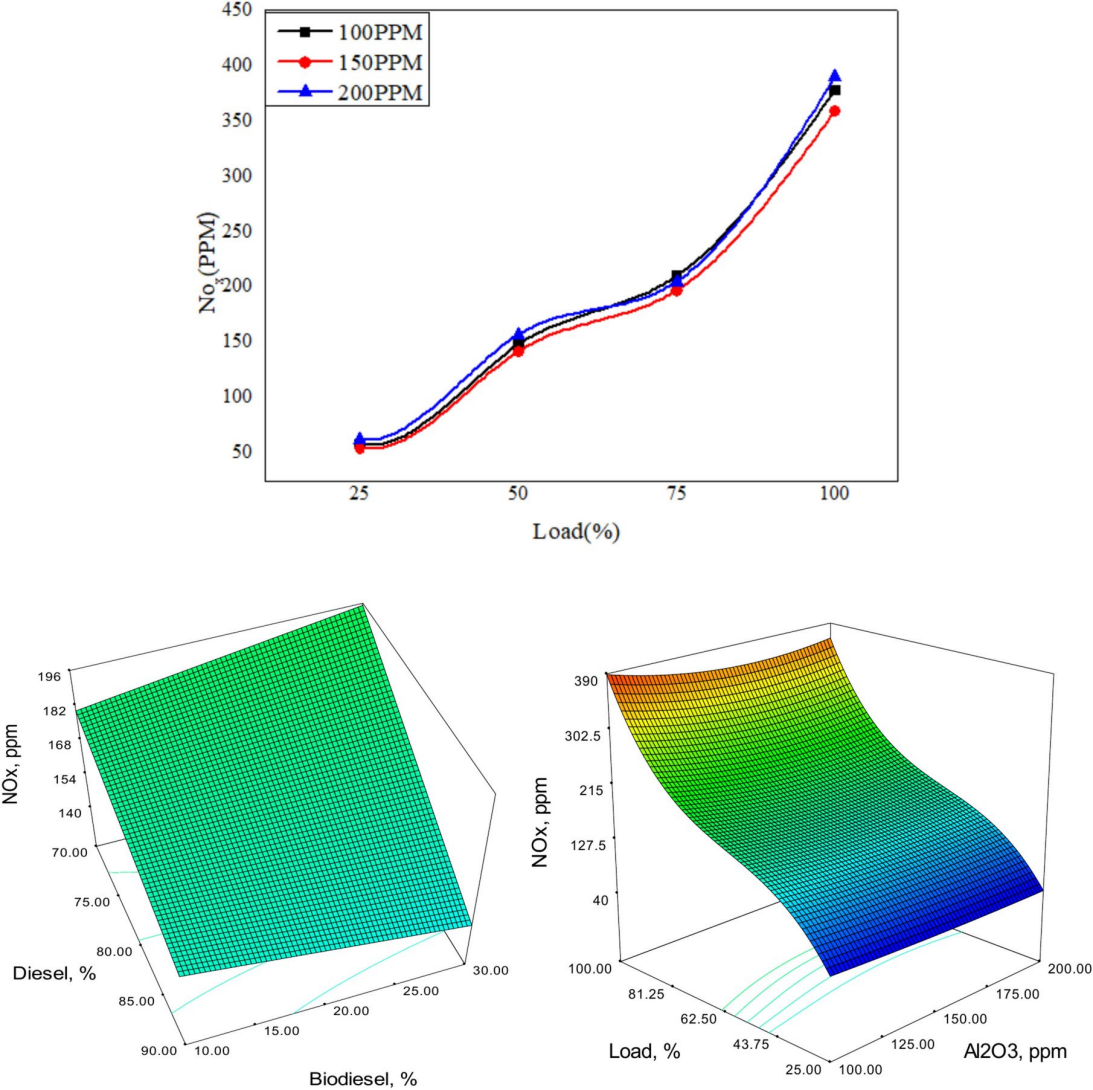
Variation of NO_x_ versus load for different blends of biodiesel blend with 90% diesel and 10% bio fuel with nano particles.

**Fig. 23:**
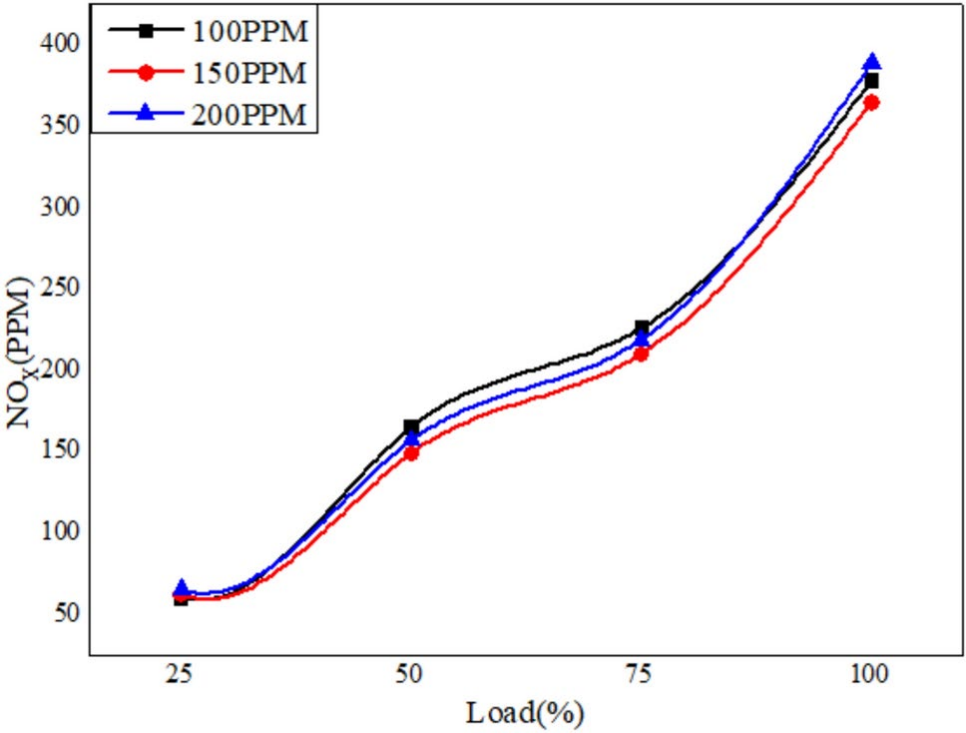
Variation of NO_x_ versus load for different blends of biodiesel blend with 80% diesel and 70% bio fuel with nano particles.

**Fig. 24:**
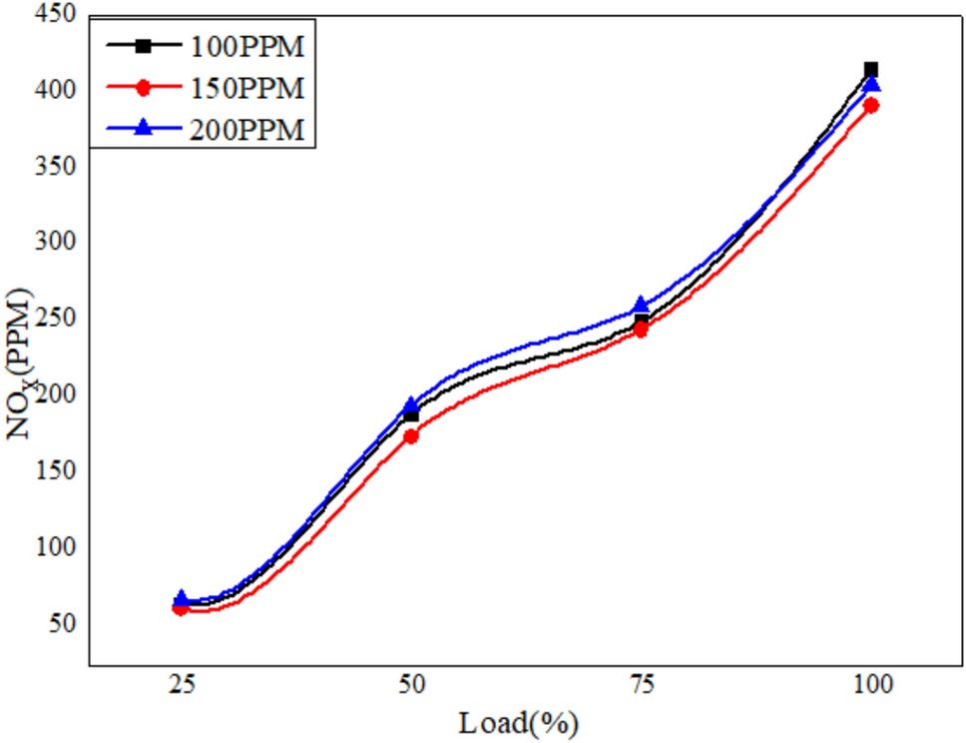
Variation of NO_x_ versus load for different blends of biodiesel blend with 70% diesel and 30% bio fuel with nano particles.

Figures 22, 23 and 24 depict the plots for the evaluation of NOx emissions against load. The graphs clearly show that the emission of nitrogen oxide for three different blends of biofuels dispersed with Nanoparticles in different proportions decreases with the increase in the nanoparticles due to better combustion of the blends in the CI engine^58^.

The reactions involved in the NO_X_ pattern is expressed by Zeldovich’s mechanism^43^, which reacts the amount of nitrogen molecules combined with the oxygen molecules at the tailpipe of the combustion chamber expressed by the Eqs. (2), (3) and (4)

2 $$O+{N}_{2}\to N+NO$$

3 $${O}_{2}+N\to 0+NO$$

4 $$O+{N}_{2}\to N+NO$$

### Mathematical models developed with RSM

5 $$\begin{aligned} {\text{BSFC }} & = 0.{\text{37 }} + 0.0{\text{67}}*{\text{A }} - 0.0{\text{75}}*{\text{C }} - 0.0{\text{31}}*{\text{D }} - 0.0{\text{32}}*{\text{A}}*{\text{B}} \\ & \quad - 0.0{\text{11}}*{\text{A}}*{\text{C }} - 0.00{\text{625}}*{\text{A}}*{\text{D }} + 0.00{\text{975}}*{\text{C}}*{\text{D }} + 0.00{\text{71}}*{\text{C}}^{{\text{2}}} \\ \end{aligned}$$

6 $$\begin{aligned} {\text{BTE }} & = {\text{ 31}}.{\text{45 }} + 0.{\text{29A }} - 0.{\text{22}}*{\text{C }} + {\text{ 6}}.{\text{5}}*{\text{D }} + 0.{\text{43}}*{\text{A}}*{\text{B}} \\ & \quad + 0.{\text{42}}*{\text{A}}*{\text{C }} + 0.{\text{93}}*{\text{A}}*{\text{D }} - {\text{ 1}}.{\text{26}}*{\text{C}}*{\text{D }} - 0.{\text{42}}*{\text{C}}^{{\text{2}}} - {\text{ 2}}.{\text{54}}*{\text{D}}^{{\text{2}}} \\ \end{aligned}$$

7 $$\begin{aligned} {\text{CO}} & = 0.{\text{24 }} - 0.0{\text{58}}*{\text{A }} - 0.0{\text{45}}*{\text{C }} - 0.0{\text{97}}*{\text{D }} - 0.0{\text{56}}*{\text{A}}*{\text{B }} - 0.00{\text{68}}*{\text{AC}} \\ & \quad + 0.0{\text{38}}*{\text{A}}*{\text{D }} - 0.00{\text{875}}*{\text{C}}*{\text{D }} + 0.0{\text{15}}*{\text{C}}^{{\text{2}}} + {\text{1}}.0{\text{8}}*{\text{D}}^{{\text{2}}} - 0.0{\text{49}} \\ \end{aligned}$$

8 $$\begin{aligned} {\text{NOx }} & = {\text{ 167}}.{\text{82 }} - {\text{ 2}}0.{\text{11}}*{\text{A }} - {\text{ 8}}.{\text{62}}*{\text{C }} + {\text{ 66}}.{\text{83}}*{\text{D }} - {\text{ 7}}.{\text{29}}*{\text{A}}*{\text{B}} \\ & \quad + 0.{\text{88}}*{\text{A}}*{\text{C }} - {\text{ 6}}.0{\text{8}}*{\text{A}}*{\text{D }} - {\text{ 3}}.{\text{53}}*{\text{C}}*{\text{ D }} + {\text{ 12}}.0{\text{4}}*{\text{C}}^{{\text{2}}} + {\text{ 33}}.{\text{12}}*{\text{D}}^{{\text{2}}} \\ \end{aligned}$$

9 $$\begin{aligned} {\text{HC }} & = {\text{ 5}}0.{\text{81 }} - {\text{ 4}}.{\text{67}}*{\text{A }} + {\text{ 1}}.{\text{33}}*{\text{C }} + {\text{ 4}}.{\text{77}}*{\text{D }} - 0.{\text{58}}*{\text{A}}*{\text{B}} \\ & \quad + 0.0{\text{63}}*{\text{A}}*{\text{C }} + 0.{\text{65}}*{\text{A}}*{\text{ D }} - {\text{ 0.}}{\text{2}}0*{\text{C}}*{\text{D }} - {\text{ 0.}}{\text{17}}*{\text{C}}^{{\text{2}}} + 0.{\text{25}}*{\text{D}}^{{\text{2}}} \\ \end{aligned}$$

Here A denotes diesel, %, B denotes biodiesel, %, C denotes Al2O3, ppm and D denotes engine load in coded terms.

### Desirability based parametric optimization

The best mix of fuel blend composition and engine running parameters was found by means of response surface methods combined with desirability-based optimizationfor enhanced performance and low emissions^59^. To strike a compromise between fuel economy, thermal efficiency, and emission characteristics, the study examined diesel–biodiesel mixes with different engine loads and concentrations of aluminum oxide nanoparticles. Maintaining a realistic desirability score, the optimization procedure sought to decrease brake-specific fuel consumption, enhance brake thermal efficiency, and lower carbon monoxide, nitrogen oxides, and hydrocarbon emissions. With the engine running at maximum load of 100% and an aluminum oxide concentration of 100 parts per million, the ideal fuel mix comprises of 89.85% diesel and 30% biodiesel (Table 8). The lowest brake-specific fuel consumption of 0.45 kg per kilowatt-hour that the optimization produced points to effective fuel use. With a little variance of 3.33%, the brake thermal efficiency was maximized at 38.18%, quite near to the validation result of 37.89%. Because of their combined effects, biodiesel and nanoparticles point to a better combustion process shown by lower fuel use and higher efficiency^60^

**Table 8:** Optimized values and parametric ranges.

Name	Goal	Lower limit	Upper limit	Optimized value	Validation results	% error
Diesel, %	Is in range	70	90	89.85	89.85	–
Biodiesel, %	Is in range	10	30	30	30	–
Load, %	Is in range	25	100	100	100	–
Al2O3, ppm	Is in range	100	200	100	100	–
BSFC, kg/kWh	Minimize	0.25	0.63	0.45	0.465	
BTE, %	Maximize	19.5	39.03	38.18	37.89	3.33
CO, %	Minimize	0.3	4	2	2.04	2
NOx, ppm	Minimize	50	400	347.83	355	2.06
HC, ppm	Minimize	39	62	49.98	51.21	2.46
Desirability				0.63		

**Fig. 25:**
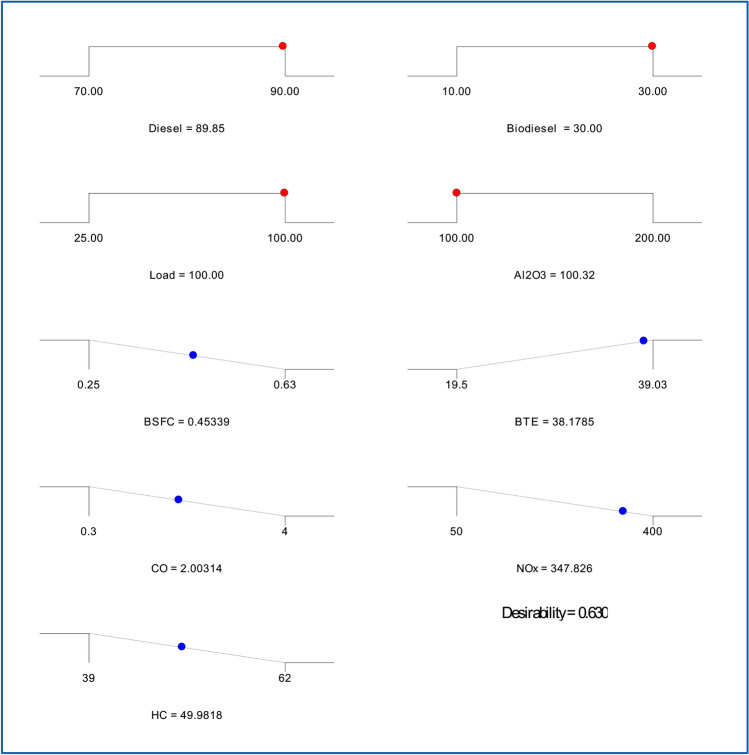
Ramp plots.

**Fig. 26:**
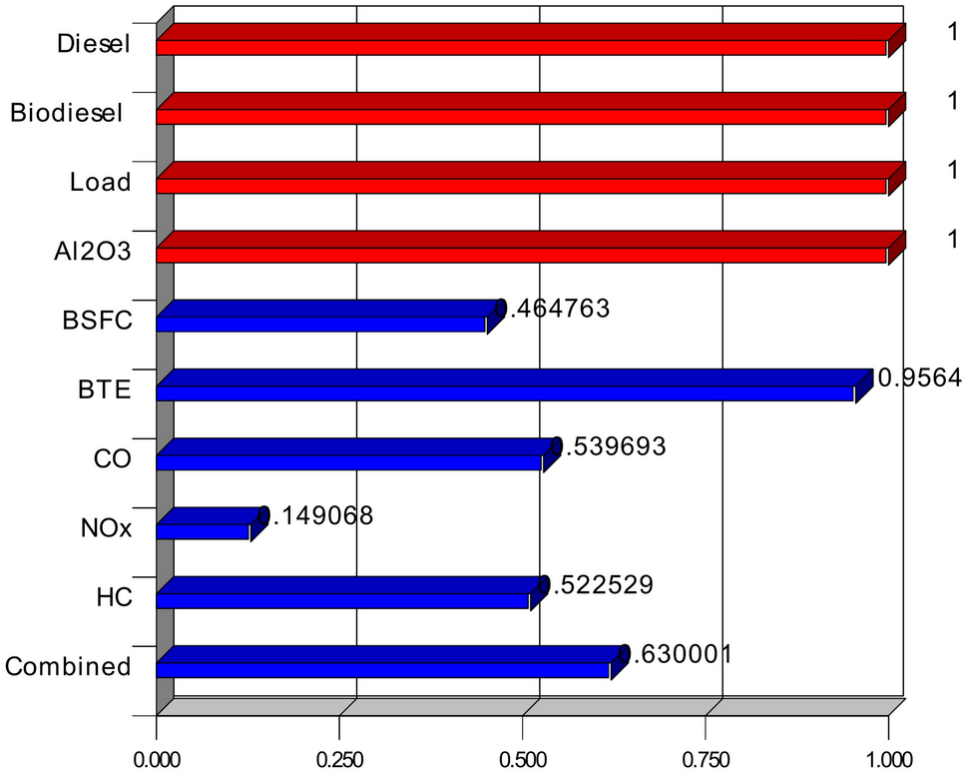
Desirability bar plots.

With carbon monoxide down to 2%, nitrogen oxides to 347.83 parts per million, and hydrocarbons to 49.98 parts per million, emission characteristics also were adjusted. Confirming the dependability of the ideal values, the validation findings revealed minimal percentage errors of 2% for carbon monoxide, 2.06% for nitrogen oxides, and 2.46% for hydrocarbons. The little discrepancies noted between the validated and optimal findings point to the response surface technique model’s resilience in underlining engine performance and emissions under the specified circumstances. With an overall desirability function value of 0.63, the consequence of well-balanced optimization guarantees a suitable trade-off between pollution control, performance improvement, and fuel economy. The findings show that by increasing combustion efficiency and lowering hazardous exhaust emissions, combining biodiesel with aluminum oxide nanoparticles can help to sustain engine running (Figs. 25, 26). Response surface methodology offers a methodical and efficient means of multi-objective optimization for engine parameter fine-tuning to reach best performance. These results show how well new fuel mixes and nano-additives could improve diesel engine efficiency while nevertheless addressing environmental issues. The method suggested in this work can be expanded to more investigation on alternate fuels and engine modifications to reach better performance criteria and compliance with pollution rules.

## Conclusions

This study thoroughly investigated the performance and emission characteristics of a single-cylinder diesel engine using Neem Oil biodiesel blended with alumina nanoparticles. Various machine learning algorithms, including Linear Regression, Decision Tree, and Random Forest, were employed to predict engine behavior based on experimental data. The following results were obtained after the investigation:

- With the engine running at maximum load of 100% and an aluminum oxide concentration of 100 parts per million, the optimal fuel mix comprises of 89.85% diesel and 30% biodiesel.
- The lowest brake-specific fuel consumption of 0.45 kg per kilowatt-hour that the optimization produced points to effective fuel use.
- With a little variance of 3.33%, the brake thermal efficiency was maximized at 38.18%, quite near to the validation result of 37.89%.
- The addition of alumina nano particles improves the performance of the engines and emission characteristics of the engine, due to increase in surface pressure and surface area available for combustion with the addition of nano particles. Nano particles also acts as catalyst to speed up, combustion process and ensures complete combustion there by reducing emission characteristics of blends under consideration.
- Exhaust gas temperature with 150 PPM dispersion of nano particles shows better results indicates better combustion efficiency and complete fuel oxidation, which can improve engine performance.
- The Exhaust gas temperature and thermal efficiency is increased due to the higher calorific value of bio-fuel and increased thermal conductivity.
- The chemical reaction with addition of nano particles releases more oxygen which further reduces emission rate by reducing CO emissions.
- Addition of nano particles slightly increases the emission of NO_x_ as a result of increase of radicals.
- The Random Forest model demonstrated the highest predictive accuracy for performance (Test R^2^ = 0.9620, Test MAPE = 3.6795%), making it the most reliable statistical approach for predicting BSFC compared to Linear Regression and Decision Tree models.
- The Random Forest model also outperformed other approaches in predicting emissions, achieving the highest accuracy with a Test R^2^ of 0.9826 and the lowest Test MAPE of 9.3067%.
- The Linear Regression and Random Forest models demonstrated strong predictive accuracy for NOx emissions, with LR achieving the highest Test R^2^ (0.9972) and RF offering a balanced trade-off between accuracy and generalizability (Test R^2^ = 0.9392, Test MAPE = 2.2773%).

From an industrial perspective, these findings have significant implications for sustainable fuel development and diesel engine optimization. The use of biodiesel with nanoparticles can serve as a viable alternative to conventional diesel, promoting cleaner combustion and improved efficiency. The application of machine learning provides robust insights into the complex relationships between fuel blends, nanoparticle concentrations, and engine performance, offering a valuable tool for optimizing biofuel usage in CI engines. The use of ML models can further assist in real-time monitoring and predictive maintenance, reducing operational costs and enhancing sustainability in the transportation and energy sectors. Future studies could explore deep learning techniques for further refinement of predictive models and real-time implementation in engine control systems.

## Data Availability

The data that supports the findings of this study are available within the article.

## Abbreviations

θ: Crank angle (°C)
BTE: Brake thermal efficiency (%)
A/F: Air fuel ratio (−)
BSFC: Brake specific fuel consumption (g/kWh)
CI: Compression ignition
CO: Carbon monoxide (Vol%)
CN: Cetane number
CR: Compression ratio
DI: Direct injection
EGT: Exhaust gas temperature (°C)
HC: Hydrocarbon (PPM)
NOx: Nitrogen oxides (PPM)
ppm: Parts per million (–)
RSM: Response surface methodology
RPM: Revolutions per minute (rev/min)
DOE: Design of experiments
